# Multi-Level Thresholding Image Segmentation Based on Improved Slime Mould Algorithm and Symmetric Cross-Entropy

**DOI:** 10.3390/e25010178

**Published:** 2023-01-16

**Authors:** Yuanyuan Jiang, Dong Zhang, Wenchang Zhu, Li Wang

**Affiliations:** 1 School of Electrical and Information Engineering, Anhui University of Science and Technology, Huainan 232000, China; 2 Institute of Environment-Friendly Materials and Occupational Health, Anhui University of Science and Technology, Wuhu 241003, China

**Keywords:** slime mould algorithm, multi-level thresholding image segmentation, symmetric cross-entropy, meta-heuristics

## Abstract

Multi-level thresholding image segmentation divides an image into multiple regions of interest and is a key step in image processing and image analysis. Aiming toward the problems of the low segmentation accuracy and slow convergence speed of traditional multi-level threshold image segmentation methods, in this paper, we present multi-level thresholding image segmentation based on an improved slime mould algorithm (ISMA) and symmetric cross-entropy for global optimization and image segmentation tasks. First, elite opposition-based learning (EOBL) was used to improve the quality and diversity of the initial population and accelerate the convergence speed. The adaptive probability threshold was used to adjust the selection probability of the slime mould to enhance the ability of the algorithm to jump out of the local optimum. The historical leader strategy, which selects the optimal historical information as the leader for the position update, was found to improve the convergence accuracy. Subsequently, 14 benchmark functions were used to evaluate the performance of ISMA, comparing it with other well-known algorithms in terms of the optimization accuracy, convergence speed, and significant differences. Subsequently, we tested the segmentation quality of the method proposed in this paper on eight grayscale images and compared it with other image segmentation criteria and well-known algorithms. The experimental metrics include the average fitness (mean), standard deviation (std), peak signal to noise ratio (PSNR), structure similarity index (SSIM), and feature similarity index (FSIM), which we utilized to evaluate the quality of the segmentation. The experimental results demonstrated that the improved slime mould algorithm is superior to the other compared algorithms, and multi-level thresholding image segmentation based on the improved slime mould algorithm and symmetric cross-entropy can be effectively applied to the task of multi-level threshold image segmentation.

## 1. Introduction

Image segmentation is a key part of image processing [1,2,3], which aims to extract the target region of interest from the image. Image segmentation is widely used in various fields, such as medical image processing [4,5], agricultural image processing [6,7], and remote sensing image analysis [8,9], because of its simplicity and effectiveness. At present, the commonly used image processing methods are threshold segmentation [10], region segmentation [11,12], and clustering segmentation [13,14]. Among them, the threshold segmentation method is a popular research direction in the field of image segmentation. For complex images, multiple thresholds are selected to segment the image into multiple effective targets.

Multi-threshold segmentation identifies a set of threshold values in the image to be segmented according to a certain criterion and segments the image into multiple parts. Common threshold selection methods for multi-threshold segmentation criteria include Otsu’s method (by maximizing the between-class variance), Kapur’s entropy method (by maximizing the entropy of the classes), fuzzy entropy, minimum cross-entropy, etc. [15,16,17]. However, the multi-threshold segmentation of images expands the search space with the increase in the number of thresholds, the computational complexity increases exponentially, and the computational efficiency is low.

In recent years, meta-heuristic algorithms (MAs) have been widely used for data processing and practical problems such as multi-peaked, high-dimensional, and nonlinear complex computations. In the face of uncertainty or a large range of solution spaces, MAs use their stochastic search capability to obtain the optimal solution without traversing the solution space, which can greatly reduce the computational effort and the optimization search time, with examples such as the seagull optimization algorithm (SOA) [18], grey wolf optimizer (GWO) [19], sparrow search algorithm (SSA) [20], moth-flame optimization (MFO) [21], pelican optimization algorithm [22], etc. Due to the superiority of MAs, researchers have applied the optimization algorithms to multilevel threshold image segmentation tasks [23]. Lang et al. [24] used differential evolution (DE) as a local search technique to improve the situation whereby WOA falls into local optimization in the later iterations and combined WOA-DE with Kapur’s entropy to obtain an effective image segmentation method. Yu et al. [25] optimized the grey wolf optimizer by modifying the weights of the first three wolves to make full use of the knowledge of the first three wolves and achieved good results in the image segmentation task. Zhao et al. [26] improved the ant colony optimization (ACO) algorithm by the randomized alternate strategy and chaotic enhancement strategy and performed image segmentation experiments at low and high threshold levels, respectively, and the experimental results were satisfactory. Houssein et al. [27] proposed an image-thresholding method based on the black widow optimization algorithm to extract the optimal threshold values for the selected images using Otsu’s and Kapur’s entropy methods, respectively. Chen et al. [28] used a multi-strategy optimized shuffled frog leaping algorithm (SFLA) combined with Kapur’s entropy method for the multi-threshold image segmentation of common breast cancers, which outperformed the other competitors in terms of the solution efficiency and time complexity. MAs show a good segmentation performance in the field of multi-threshold image segmentation. The slime mould algorithm (SMA) [29] is a newly proposed meta-heuristic swarm intelligence algorithm with the advantages of a high merit-seeking ability and few parameters. However, in the late iterations of the algorithm, SMA, like other intelligent algorithms, is prone to fall into the local optimal solution. Örnek et al. [30] proposed an enhanced slime mould algorithm (ESMA) with a better ability to avoid local optimization by updating the position of the sticky bacterium using sine and cosine trigonometric functions. Hu et al. [31] proposed a dispersed foraging SMA (DFSMA) with a dispersed foraging strategy, which outperformed the other optimizers in terms of the convergence speed and accuracy.

In this paper, we propose an improved slime mould algorithm, called ISMA, for the multilevel thresholding image segmentation task. First, elite opposition-based learning (EOBL) was used to improve the quality and diversity of the initial population and accelerate the convergence speed. The adaptive probability threshold was used to adjust the selection probability of the slime mould so as to enhance the ability of the algorithm to jump out of the local optimum. The historical leader strategy, which selects the optimal historical information as the leader for the position update, was found to improve the convergence accuracy. Moreover, the optimization ability and solution accuracy of ISMA were verified through single-peak and multi-peak benchmark test functions. ISMA was combined with symmetric cross-entropy multi-threshold segmentation to solve the problems of the complicated calculation and low computational efficiency of multi-threshold image segmentation and realize multi-threshold image segmentation. We selected eight grayscale images as the reference images and performed a comparison of the different segmentation criteria and different MAs. The experimental results demonstrated that multi-level thresholding image segmentation based on ISMA and symmetric cross-entropy outperforms the other methods in terms of the PSNR, SSIM, and FSIM and showed significant improvements in the convergence speed and segmentation accuracy.

The main contributions of this paper can be summarized as follows:

We optimized SMA through multiple strategies and propose an improved SMA, called ISMA;

We evaluated the performance of ISMA through single-peak and multi-peak benchmark test functions;

We combined ISMA with symmetric cross-entropy for threshold segmentation and compared it with Otsu’s and Kapur’s entropy methods and minimum cross-entropy;

We compared the performance of ISMA with other MAs and evaluated the image segmentation quality through PSNR, SSIM, and FSIM.

The rest of the paper is organized as follows. Section 2 introduces the slime mould algorithm. Section 3 introduces the improvement strategy for the slime mould algorithm, elite opposition-based learning, the adaptive probability threshold, and the historical leader. Section 4 discusses ISMA performance tests. Section 5 introduces the threshold segmentation technology combining ISMA and symmetric cross-entropy. Section 6 describes image segmentation tests and analyzes the experimental results. Finally, Section 7 summarizes this paper and provides the future research directions.

## 2. Slime Mould Algorithm

The slime mould algorithm (SMA) establishes a mathematical model based on the foraging behavior of physarum polycephalum, which adjusts its position by oscillating reactions to search for the optimal food position.

The slime mould approach the food according to the odor in the air, and some individuals separate in order to search for higher-quality food in other domains after identifying the food source, as shown in the following equation:

(1) $$\vec{X(t+1)}=\{\begin{array}{c} rand\cdot (UB-LB)+LB,rand<z\\ \begin{array}{c} \vec{X_{b}(t)}+\vec{vb}\cdot \left(\vec{W}\cdot \vec{X_{A}(t)}-\vec{X_{B}(t)}\right),r<p\\ \vec{vc}\cdot \vec{X(t)},r\geq p \end{array} \end{array}$$

where $\vec{\mathrm{vb}}\;\in \;[-a,\;a]$ obeys a uniform distribution and simulates the degree of learning of the slime individuals from other individuals in the population; $\vec{\mathrm{vc}}$ simulates the degree of information retention of the slime individuals themselves, which decreases linearly from 1 to 0; *t* denotes the current iteration; $\vec{X_{b}}$ represents the position of the individual with the highest current food concentration, which is the global optimal solution; $\vec{X}$ represents the position of the slime mould; $\vec{X_{A}}$ and $\vec{X_{B}}$ denote the random individuals; $\vec{W}$ represents the weight of the slime mould; UB and LB are the upper and lower bounds of the search space; *rand* and *r* denote the random value in [0, 1]; and *z* is the weight of the separated part of the individual, which is 0.03.

The *p*, $\vec{\mathrm{vb}}$, and $\vec{W}$ can be calculated as follows:

(2) p=tanh|S(i)−DF|

(3) $$a=\mathrm{arctanh}(-(\frac{t}{\max\_t})+1)$$

(4) $$\vec{W(SmellIndex(i))}=\{\begin{array}{c} 1+r\cdot \log(\frac{bF-S(i)}{bF-wF}+1),condition\\ 1-r\cdot \log(\frac{bF-S(i)}{bF-wF}+1),others \end{array}$$

(5) SmellIndex=sort(S)

where *i* ∈ 1, 2, …, *n*, *S*(*i*) denotes the fitness value of the slime mould; *DF* denotes the optimal fitness value; the condition is the rank first-half fitness of the search agent of *S*(*i*); *r* denotes the random value in [0, 1]; max_*t* denotes the maximum iteration; *bF* and *wF* denote the best fitness and the worst fitness currently obtained, respectively; and *SmellIndex* denotes the slime mould individuals sorted by fitness.

The $\vec{W}$, $\vec{\mathrm{vb}}$, $\vec{\mathrm{vc}}$ in the slime mould algorithm are used to simulate the oscillatory response of the slime mould so that the slime mould can approach in order to grasp quality food faster; $\vec{\mathrm{vb}}$ oscillates randomly between [−*a*, *a*], gradually approaching zero; and $\vec{\mathrm{vc}}$ oscillates between [−1, 1] and eventually tends toward zero.

## 3. The Proposed Method

### 3.1. Elite Opposition-Based Learning

The population initialization of the SMA randomly generates the population positions, which causes the population to have large randomness and uncertainty and affects the final convergence speed and accuracy. Opposition-based learning introduces the reverse solution, which effectively increases the diversity and quality of the algorithm population. However, the reverse solution generated by the reverse learning may render it more difficult to search for the optimal value than the current search space. Elite opposition-based learning takes advantage of elite individuals carrying more effective information compared with ordinary individuals so as to improve the diversity and population quality of the mucilage population and enhance the global search performance and convergence accuracy of the algorithm. In this paper, we apply EOBL to the initialization of SMA, take advantage of the feature according to which elite individuals contain more valid information with which to construct inverse populations, and select the better individuals from the current population and the inverse population as the initial solution so as to improve the quality and diversity of the initial populations.

Assuming that elite individuals $X_{i,j}{\;=(x}_{i,1},x_{i,2},\cdots {,x}_{i,d})(i=1,2,\cdots ,N;\;j=1,2,\cdots ,d)$, the inverse solution $\bar{X_{i,j}}=\bar{x_{i,1}},\bar{x_{i,2}},\cdots ,\bar{x_{i,d}}$ is defined as follows:

(6) $$\bar{X_{i,j}}=K*(\alpha_{j}+\beta_{j})-X_{i,j}$$

where the dynamic coefficient K ∈ (0,1),$\;X_{i,j}\in \left[\alpha_{j},\beta_{j}\right]$,${\;\alpha }_{j}{=\min(X}_{i,\;j})$,$\;\beta_{j}{=\max(X}_{i,\;j})$, *α_j_*_,_ *β_j_* denotes the dynamic boundary. The dynamic boundary overcomes the disadvantage of the difficulty in preserving the search experience at the fixed boundary, so that the elite inverse solution can be located in a narrow search space, which is conducive to the convergence of the algorithm. If the dynamic boundary operation causes $\bar{X_{i,\;j}}$ to cross the boundary and become an infeasible solution, it will be reset using the random generation method in the following way:

(7) $$\bar{X_{i,j}}=\mathrm{rand}(\alpha_{j},\beta_{j})$$

### 3.2. The Adaptive Probability Threshold

The SMA balances the different movements of the slime mould surrounding the food and grasping food by the adaptive parameter p. During the iteration, when the current individual fitness differs significantly from the optimal fitness, *p* is 1, and the slime mould individual updates its position using the movement of surrounding the food. However, when the value range of the test function is small, it will probably update the position by the movement method of grasping food, and it will be impossible to choose a predatory strategy that is suitable for the current slime mould population, causing a slow convergence and low accuracy. Therefore, this paper introduces a new adaptive probability threshold to cause the slime mould to select the appropriate predation strategy for the current population, thus improving the convergence speed. The adaptive probability threshold mathematical model is described as shown in Equation (8):

(8) $$p=\tanh(\frac{10\cdot |bF-S(i)|}{|bF-wF|+\varepsilon })$$

where *ε* is the minimum constant preventing the denominator from being zero.

### 3.3. The Historical Leader

During the search process of SMA, the update of the ith slime mould concentration at the *t*+1 iteration mainly depends on the best global slime mould concentration at the current iteration number t, resulting in an insufficient global search, rendering it easy to fall into the local extreme value region, and, sometimes, causing the low convergence accuracy of the algorithm. In this paper, the first- and second-best positions of the previous generation and the global optimal position are introduced as leaders in the slime mould position update formula, and the magnitude and direction of the slime mould surrounding the food are controlled according to the optimal historical information and current state, a method which effectively avoids the problem of the basic algorithm’s tendency to easily fall into the local extreme value region and improves the algorithm’s search accuracy. The ISMA location update formula is as follows:

(9) $$\vec{X(t+1)}=\{\begin{array}{c} rand\cdot (UB-LB)+LB,rand<z\\ \begin{array}{c} \vec{X_{C}(t)}+\vec{vb}\cdot \left(\vec{W}\cdot \vec{X_{D}(t)}-\vec{X_{E}(t)}\right),r<p\\ \vec{vc}\cdot \vec{X(t)},r\geq p \end{array} \end{array}$$

where $\vec{X_{C}}$ is the current global optimal position, and $\vec{X_{D}}$ and $\vec{X_{E}}$ are the first- and second-best positions of the previous generation, respectively.

### 3.4. Pseudo-Code of ISMA

The pseudo-code of ISMA is shown in Algorithm 1.
**Algorithm 1** Pseudo-code of ISMAInitialize the parameters *popsize*, *Max_iteraition*Initialize the positions of the slime mould**While** (*t* ≤ *Max_iteraition*)    Calculate the fitness of all the slime mould    *Update bestFitness*, *X_b_*    Calculate the *W* by **Equation** (4)    **For** each search portion,        *Update p by* **Equation** (8)        *Update positions by* **Equation** (9)        
**End For**
    *t* = *t* + 1**End While****Return** *bestFitness*, *X_b_*

## 4. ISMA Performance Evaluation Experiments and Analysis

The experiments were conducted on a computer with Intel(R) Core(TM) i7-10870H CPU @ 2.20 GHz, 8 GB of RAM, the Windows10 operating system, and MATLAB 2020a compiler software.

**Figure 1:**
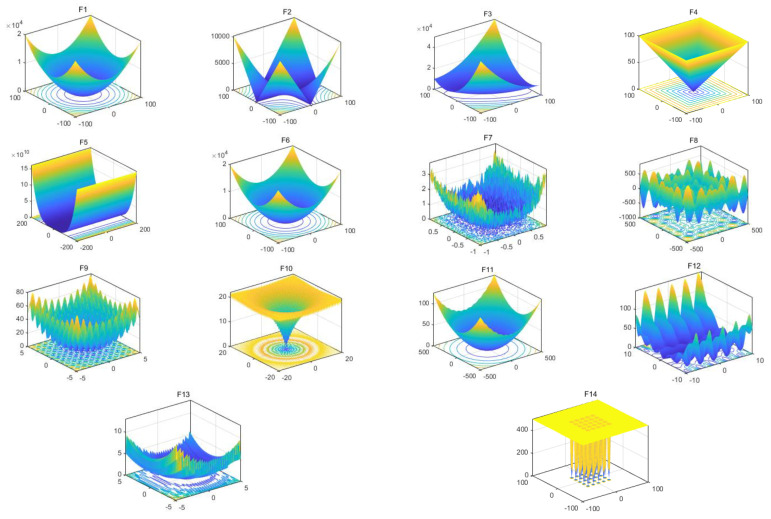
View of the 14 benchmark functions.

In order to test the optimization ability and solution accuracy of ISMA, 14 benchmark functions [32] were selected to test the algorithm’s performance. The information of the benchmark test functions is shown in Table 1 which are divided into single-peak functions (F1–F7) and multi-peak functions (F8–F14). D, UM, and MM denote the function dimension, single-peak function, and multi-peak function, respectively. The function visualization is shown in Figure 1. These functions are defined in Table 1. It can be seen that the single-peak function has only one global optimal solution, which can verify the search ability of the algorithm. The multi-peak function has many local optimal solutions and only one global optimal solution, which can verify the escape ability of the local optimal solution of the algorithm.

To verify the performance of the proposed ISMA, it was compared with seven other algorithms, including SMA, SOA, MFO, POA, GWO, ESMA, and DFSMA. Table 2 illustrates the parameter settings of each algorithm. For all the algorithms in the comparison, the population size *n* = 50 and the maximum number of iterations max_*t* = 500. Due to the fact that the search strategy of the MAs is random, it was run 30 times independently on all the benchmark functions, respectively, and the evaluation criteria were the average fitness (mean), standard deviation (std), and computation time. The experimental results are provided in Table 3, Table 4 and Table 5, where the best values are marked in bold.

As can be seen from the table analysis, the statistical results of ISMA for the 14 test functions are significantly better than those of the other four comparison algorithms under the same constraints. Among the single-peak functions, for F1, F2, F3, and F4, ISMA can identify the theoretical optimal solution in all 30 experiments, while SOA, MFO, and GWO have larger mean values, and SMA, POA, ESMA, and DFSMA have smaller mean values and are closer to the optimum. However, the std values of ISMA are all 0. Only SMA, ESMA, and DFSMA obtain std values of 0 for F1 and F3. For F5, F6, and F7, none of the algorithms obtain the optimal values stably, but ISMA obtains mean values closer to the optimal solution, with smaller std values. In the multi-peak function, for F8, F9, F11, and F14, ISMA achieves the theoretical optimal value with the smallest std value, ESMA and DFSMA achieve the mean values close to the std value, and SMA is slightly larger than ESMA and DFSMA. For F10, F12, and F13, none of the algorithms achieve the optimal solution stably, but ISMA achieves a better performance. The analysis of the experimental results shows that ISMA outperforms the other comparison algorithms in relation to the 14 benchmark functions tested.

The average CPU times of the different algorithms in the 14 benchmark functions are shown in Table 5. As can be seen from the table, ISMA takes a relatively longer time to compute; however, ISMA can still outperform some algorithms with less time spent, such as ESMA and DFSMA. In general, ISMA still has a great advantage over the other algorithms.

**Figure 2:**
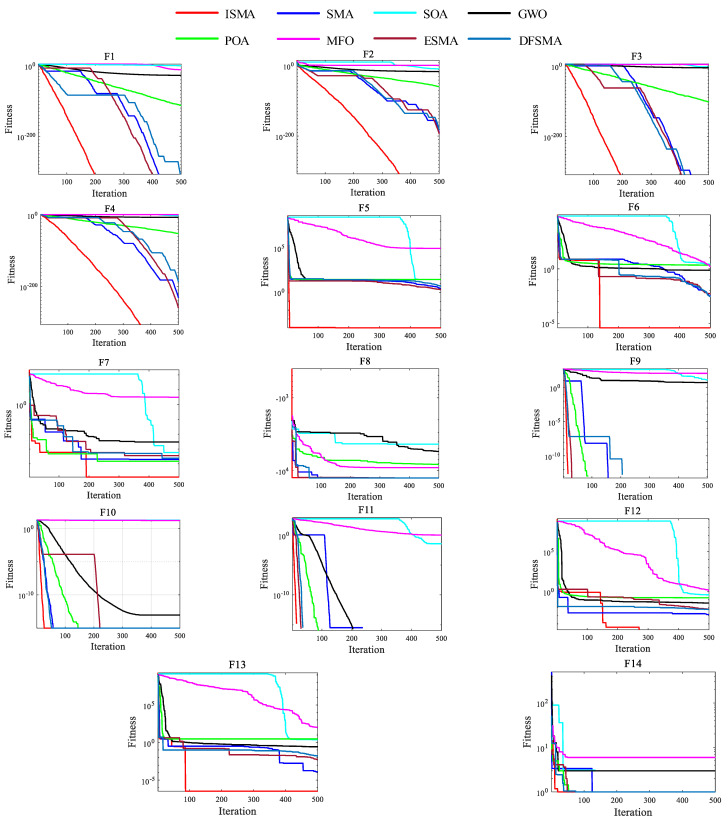
Convergence behavior of the algorithms based on 14 benchmark functions.

To reflect the dynamic convergence characteristics of ISMA, the convergence curves of seven optimization algorithms under 14 benchmark functions are shown in the Figure 2. For F1, F2, F3, F4, F8, F9, F10, and F11, ISMA is obviously superior to the other algorithms in terms of the convergence speed and optimization accuracy, and the search performance in the early iteration and the exploitation performance at the end of the iteration are also superior to those of the other algorithms. This shows that EOBL causes ISMA to ensure the exploitation ability and the search ability without losing the population diversity and search stability. For F5, F6, F7, F8, F12, F13, and F14, with the increase in the iterations, various algorithms stalled to different degrees and fell into local optimum. However, due to the introduction of the adaptive threshold, ISMA could effectively jump out of local optimum and obtain a better search accuracy.

In summary, whether single-peak or multi-peak functions are applied, ISMA shows a better overall search performance and a better solution accuracy and stability than the seven representative comparison algorithms, with a superior solution performance. It was shown that ISMA can explore the search space sufficiently and efficiently and ensure the global search capability and local exploration capability. ISMA solves the problem of the susceptibility of the SMA algorithm to fall into the local extreme value region, with an unstable optimization performance and low precision, when solving complex functions.

## 5. Multi-Threshold Segmentation

### 5.1. Symmetric Cross-Entropy Threshold Segmentation

In 1968, Kullback proposed cross-entropy for the measurement of the difference in information between two probability distributions [33]. Let $P=\left\{p_{1},p_{2},\ldots ,p_{n}\right\}$ and $Q=\left\{q_{1},q_{2},\ldots ,q_{n}\right\}$ be two probability distributions defined based on the same set of values. The cross-entropy between *P* and *Q* can be calculated as follows:

(10) $$D(P,Q)=\sum_{i=1}^{N}p_{i}\log\frac{p_{i}}{q_{i}}$$

Multi-level threshold segmentation identifies a set of thresholds in the image to be segmented according to a certain criterion and segments the image into multiple parts. The minimum cross-entropy algorithm determines the threshold value by minimizing the cross-entropy between the original image and the threshold image [34].

In this paper, we use symmetric cross-entropy to determine the threshold values. Symmetric cross-entropy takes into account both gray-level and neighborhood average gray-level information and provides better results for the segmentation of real images [35]. Let the original image be *I* and *h*(*i*), i=1, 2, …, L be the corresponding histogram, with *L* being the number of grey levels. Assuming that *t* thresholds need to be selected, the object function of symmetric cross-entropy can be defined as:

(11) $$H(t)=H_{0}+H_{1}+,\ldots +H_{t}$$

where:

$$H_{0}=\sum_{i=0}^{t}h_{i}(i\ln\frac{i}{u_{0}(t)}+u_{0}(t)\ln\frac{u_{0}(t)}{i})$$

$$H_{1}=\sum_{i=1}^{t}h_{i}(i\ln\frac{i}{u_{1}(t)}+u_{1}(t)\ln\frac{u_{1}(t)}{i})$$

$$H_{t}=\sum_{i=t+1}^{L-1}h_{i}(i\ln\frac{i}{u_{t}(t)}+u_{t}(t)\ln\frac{u_{t}(t)}{i})$$

Above, $H_{0}$,${\;H}_{1}$,…,${\;H}_{n}$ denote the entropies of distinct classes.

In order to obtain the optimal threshold values, the fitness function in Equation (12) is minimized:

(12) $$t(1,\ldots ,n)*=\underset{0\leq t\leq L-1}{\arg\min}\{H_{0}+H_{1}+,\ldots +H_{t}\}$$

### 5.2. Multi-Level Threshold Segmentation Based on ISMA and Symmetric Cross-Entropy

In order to improve the accuracy and computational speed of the multi-threshold segmentation technique, multi-level threshold segmentation based on ISMA and symmetric cross-entropy is proposed. The method determines the optimal threshold value by minimizing the objective function given in Equation (12). The steps are as follows:

(a) Read the image to be segmented (grayscale image).
(b) Find the grayscale histogram of the image.
(c) Initialize the parameters of ISMA, the size of the population of slime mould (*n*), the maximum number of iterations (max_*t*), the initial values of the upper bound (LB) and lower bound (UB), and the number of desired partition thresholds (*d*).
(d) Find the optimal fitness value using symmetric cross-entropy as the ISMA objective function.
(e) If ISMA reaches the maximum number of iterations max_*t*, the optimization is completed, and the slime mould location information regarding the best fitness is returned, which is the best segmentation threshold. Otherwise, skip to step (*d*).
(f) Perform grayscale image segmentation with the best threshold and obtain the segmented image.

## 6. Threshold Segmentation Experiment Results and Analysis

### 6.1. Threshold Segmentation Experiment for the Segmentation Criteria

**Figure 3:**
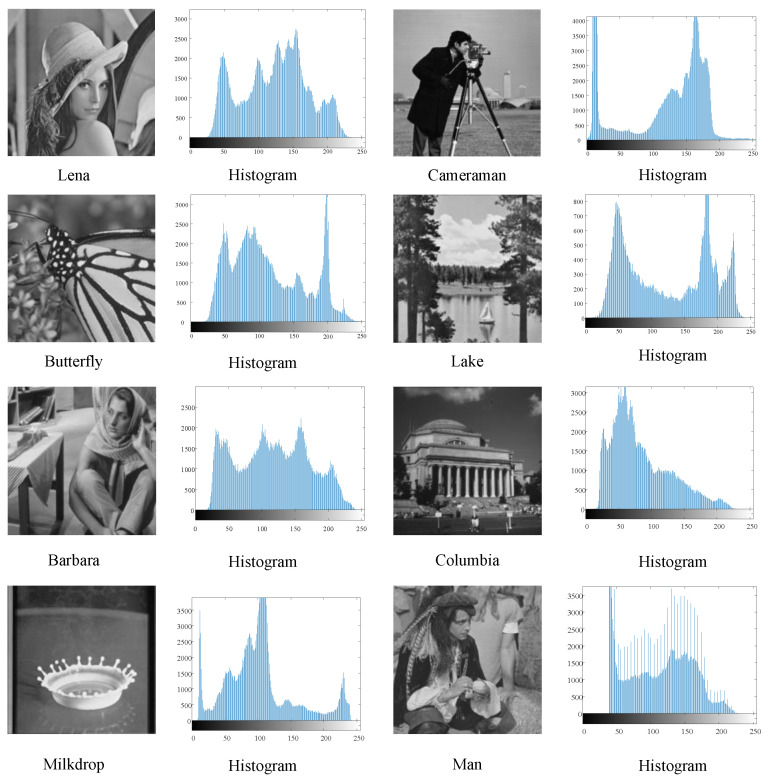
Test images and their grayscale histograms.

To verify the effectiveness of symmetric cross-entropy threshold segmentation, Lena, Cameraman, Butterfly, Lake, Barbara, Columbia, Milkdrop, and Man, the classic threshold segmentation images, were selected as the test images to test the segmentation effect of this paper’s algorithm, and the four segmentation criteria based on Otsu, Kapur’s entropy, minimum cross-entropy, and symmetric cross-entropy were compared. Here, the eight benchmark images are grayscale. These images and their histograms are presented in Figure 3. The experimental parameters of the algorithm are set as follows: the population size *n* = 50, the maximum number of iterations max_*t* = 100, the upper and lower bounds of the individuals are taken as [0, 255], and the dimension (*d*) is taken as 2, 3, 4, and 5, corresponding to 2, 3, 4, and 5 thresholding, respectively. Figure 3 shows the grayscale histograms of the eight selected images, and it can be seen that they have different histogram distributions and can represent different types of complex, multi-target images.

In order to objectively evaluate the stability of the segmentation algorithm and the effect of multi-threshold image segmentation, each image was run 30 times independently, and the peak signal to noise ratio (PSNR), structural similarity (SSIM), and feature similarity (FSIM) were selected as the evaluation criteria. PSNR was used to evaluate the image degradation, according to which the larger the value is, the smaller the image degradation and the better the image segmentation effect will be. SSIM evaluates the similarity between images based on the image brightness, contrast, and structure information. The SSIM value range is [0, 1], and the larger the value is, the more similar the image after threshold segmentation will be to the original image, and the better the image segmentation effect will be. FSIM uses image gradient features and phase consistency features for image quality evaluation, and the larger the value is, the better the image segmentation quality will be.

PSNR is computed by the following equation:

(13) $$PSNR=20\log_{10}\frac{255}{RMSE}$$

(14) $$RMSE=\sqrt{\frac{\sum_{i=1}^{M}\sum_{j=1}^{N}\left(x(i,j)-y{\left(i,j\right)}^{2}\right)}{M\times N}}$$

where *x* and *y* denote the original and segmented images, respectively. *M* and *N* are the sizes of the images.

SSIM is computed by the following equation:

(15) $$SSIM(x,y)=\frac{\left(2\mu_{x}\mu_{y}+C_{1})(2\sigma_{x}\sigma_{y}+C_{2}\right)}{\left(\mu_{x}{}^{2}+\mu_{y}{}^{2}+C_{1})(\sigma_{x}{}^{2}+\sigma_{y}{}^{2}+C_{2}\right)}$$

where $\mu_{x}$ and $\mu_{y}$ indicate the mean intensities of the original and segmented images, respectively. $\sigma_{x}$ and $\sigma_{y}$ are the standard deviations of original and segmented images. *C*_1_ and *C*_2_ are two constants equal to 0.065.

FSIM is computed by the following equation:

(16) $$FSIM=\frac{\sum_{\omega \in \Omega }S_{PC}(\omega )S_{G}(\omega )PC_{m}(\omega )}{\sum_{\omega \in \Omega }PC_{m}(\omega )}$$

(17) $$S_{PC}(\omega )=\frac{2PC_{1}(\omega )PC_{2}(\omega )+C_{3}}{PC_{1}{}^{2}(\omega )+PC_{2}{}^{2}(\omega )+C_{3}}$$

(18) $$S_{G}(\omega )=\frac{2G_{1}(\omega )G_{2}(\omega )+C_{4}}{G_{1}{}^{2}(\omega )+G_{2}{}^{2}(\omega )+C_{4}}$$

where Ω indicates the entire domain of the image. *C*_3_ and *C*_4_ are constants which are equal to 0.85 and 160, respectively. *G* indicates the gradient magnitude of an image, and PC denotes the phase congruence.

ISMA was combined with Otsu, minimum cross-entropy, Kapur’s entropy, and symmetric cross-entropy, respectively, and the experimental results are shown in Table 6. It can be seen from the results in Table 6 that a significant difference in the image quality is obtained according to the different image segmentation criteria. The quality of the images is gradually enhanced with the increase of the number of thresholds in the segmentation results, and the PSNR, SSIM, and FSIM values are gradually increased, and the image segmentation performance is gradually enhanced. Among the values, the PSNR, SSIM, and FSIM obtained from the images segmented by Kapur’s entropy thresholding at the low threshold (*d* = 2, 3) are the lowest, and the PSNR, SSIM, and FSIM values obtained from the images segmented by symmetric cross-entropy, Otsu, and minimum cross-entropy thresholding have little difference, which proves the feasibility of symmetric cross-entropy image segmentation. At the high threshold (*d* = 4, 5), the symmetric cross-entropy method outperforms Otsu, minimum cross-entropy, and Kapur’s entropy segmentation methods in terms of the PSNR, SSIM, and FSIM, proving that the results of the multi-threshold image segmentation based on symmetric cross-entropy have less distortion and retain the feature information of the original image in a more complete manner.

Table 7 denotes the best threshold values obtained according to the different segmentation criteria. It can be seen from the results in Table 7 that the optimal threshold obtained by symmetric cross-entropy differs less from that obtained by minimum cross-entropy and more from those obtained by Otsu and Kapur’s entropy at the low thresholds (2, 3). At the high thresholds (4, 5), the optimal thresholds obtained by the different image segmentation criteria are significantly different, resulting in different image qualities.

**Figure 4:**
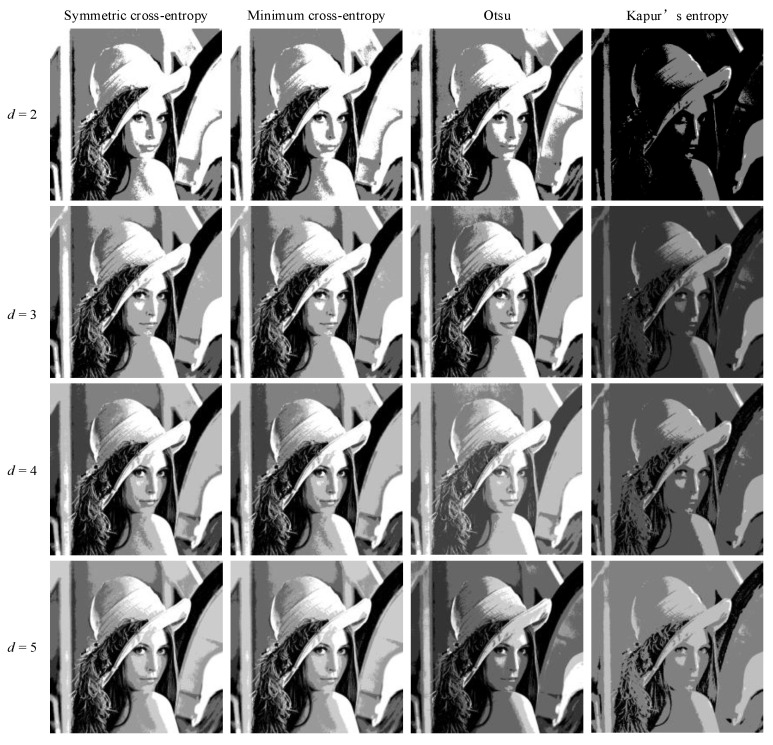
Lena image segmentation results.

**Figure 5:**
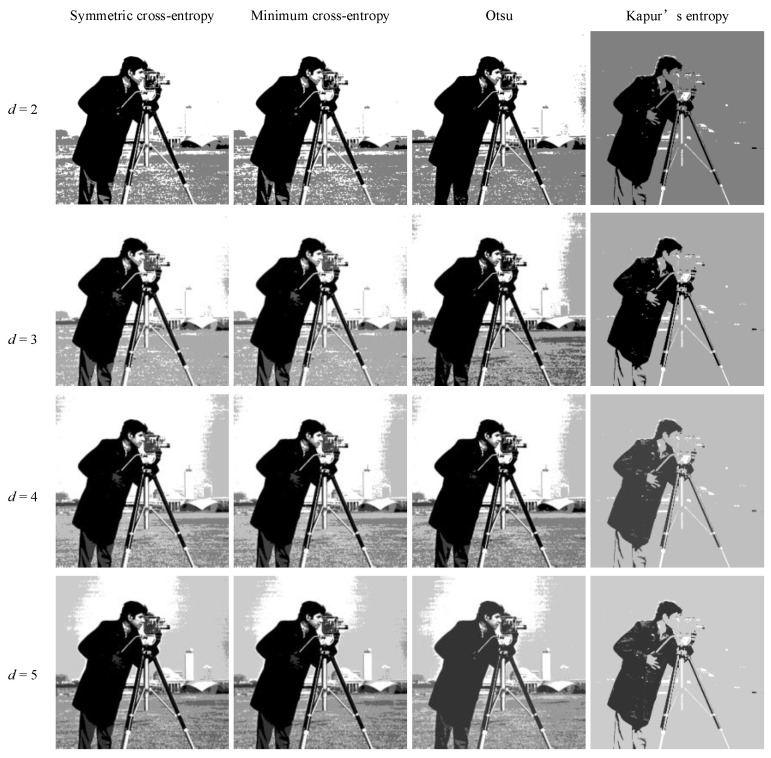
Cameraman image segmentation results.

**Figure 6:**
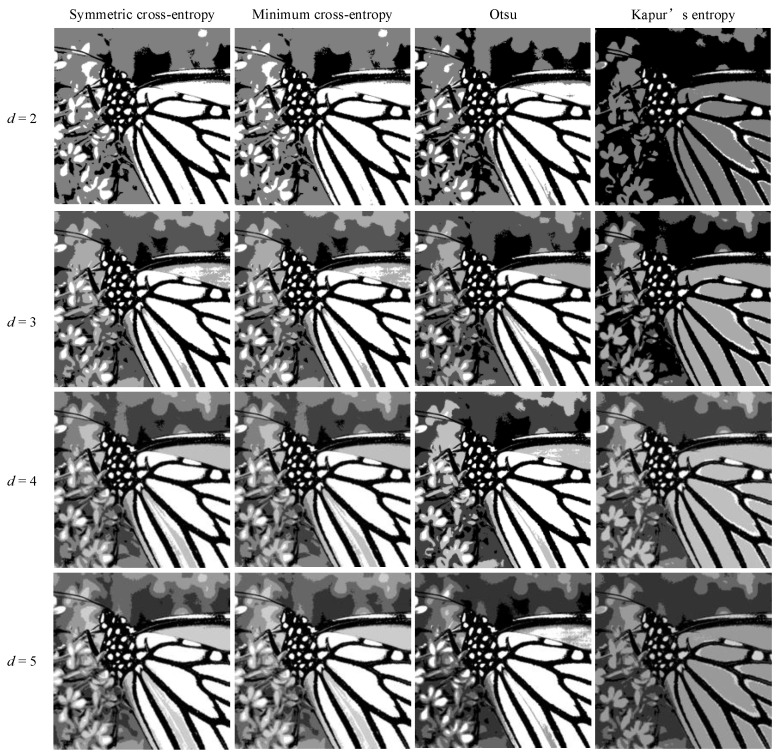
Butterfly image segmentation results.

**Figure 7:**
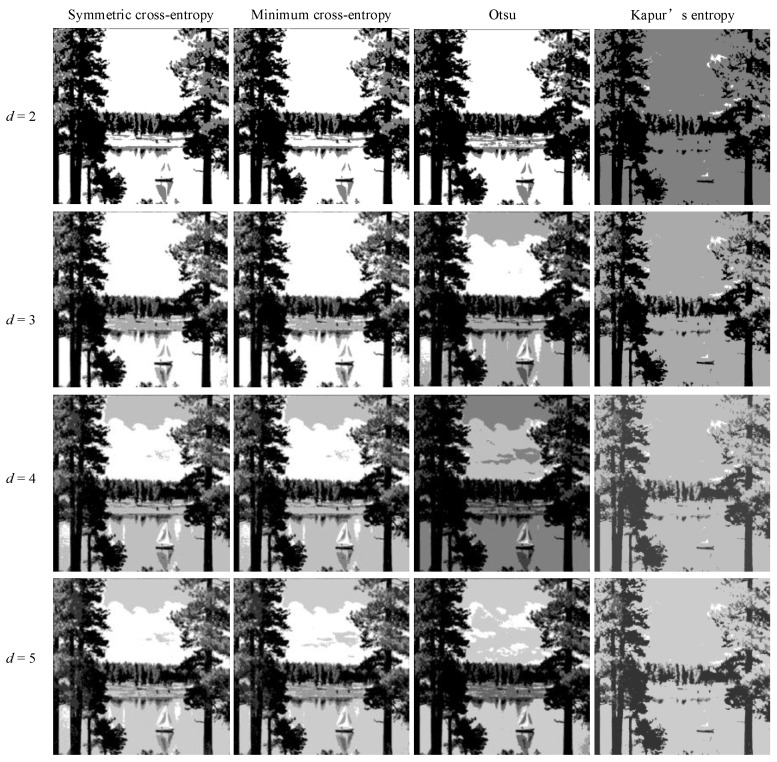
Lake image segmentation results.

**Figure 8:**
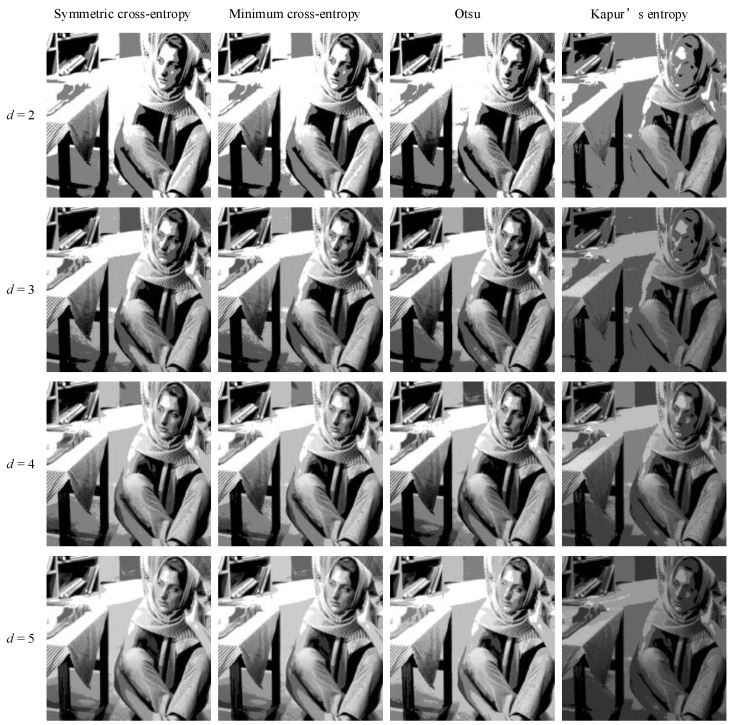
Barbara image segmentation results.

**Figure 9:**
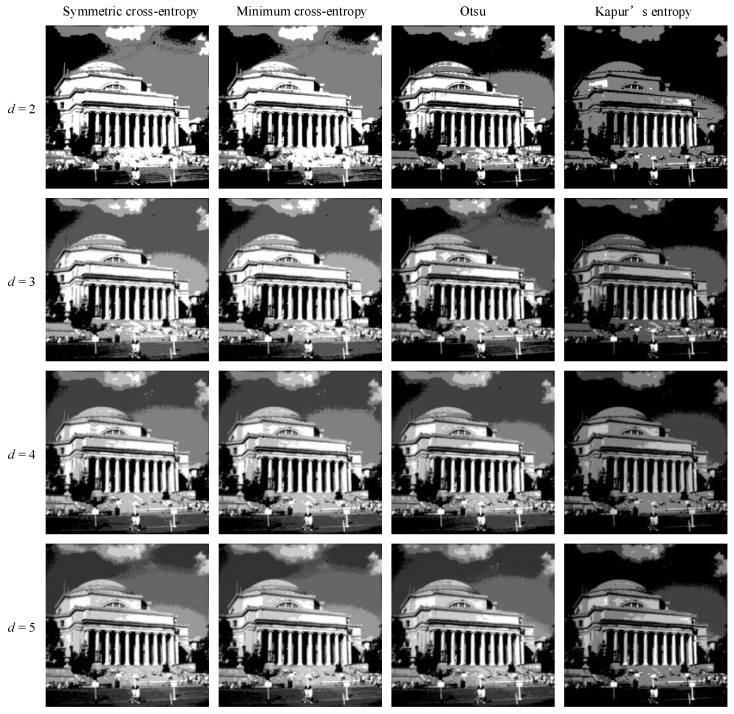
Columbia image segmentation results.

**Figure 10:**
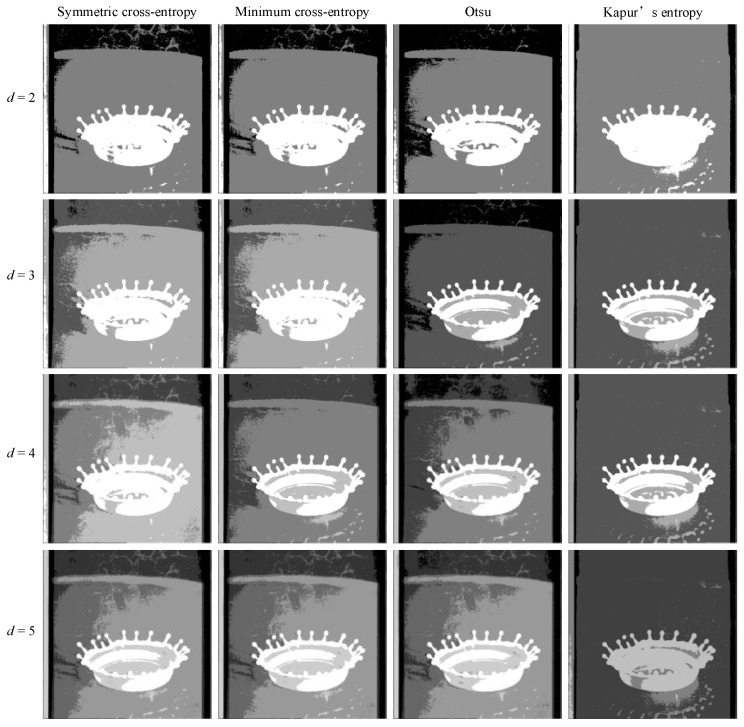
Milkdrop image segmentation results.

**Figure 11:**
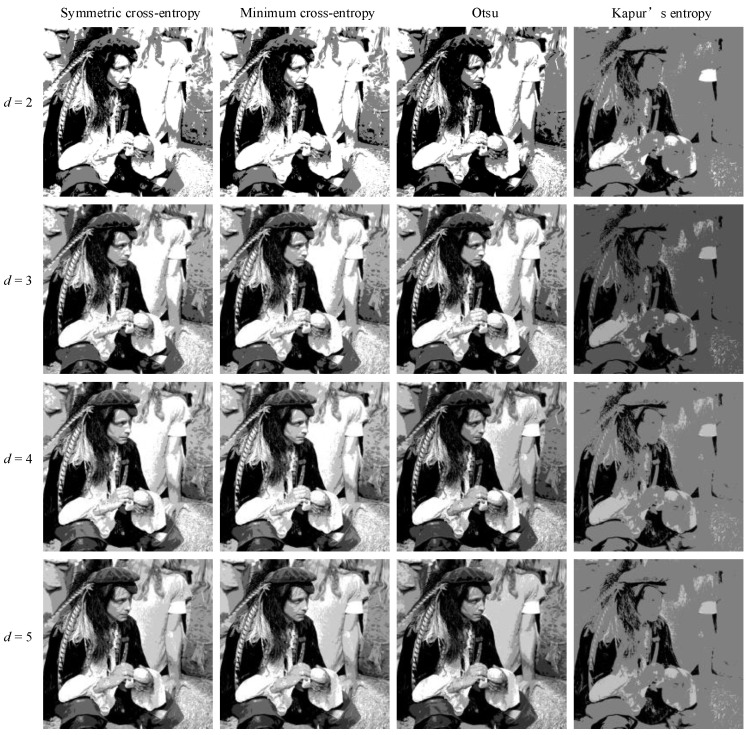
Man image segmentation results.

In order to intuitively understand the effect of multi-level threshold image segmentation, the image segmentation results of the four images based on the four segmentation criteria are shown in Figure 4, Figure 5, Figure 6, Figure 7, Figure 8, Figure 9, Figure 10 and Figure 11, respectively. From the segmented images, it can be seen that with the increase in the number of thresholds, the details of the image segmentation results are clearer, the information is more complete, and the segmentation quality is higher. When Kapur’s entropy is used as the ISMA objective function for image threshold segmentation, some information is lost in the segmentation results, and details such as the character outline are blurred, resulting in a poor segmentation effect. When symmetric cross-entropy, minimum cross-entropy, and Otsu are used as the ISMA objective function, respectively, the facial contour and background information of the person can become clearly segmented. When the thresholds are 2, 3, and 4, the segmentation effects of the three objective functions are similar, and there is almost no difference. When the threshold is 5, the image segmentation results of minimum cross-entropy and Otsu lose part of the image information and appear distorted, while the segmentation results of symmetric cross-entropy still obtain a clearer image and can provide a more complete target region, which proves the superiority of symmetric cross-entropy as the objective function.

In summary, the image quality obtained by image segmentation using symmetric cross entropy as the objective function of ISMA is better than those of the other segmentation criteria, as this method can obtain clearer images and retain more original image information.

### 6.2. Threshold Segmentation Experiment of MAs

To verify the performance of ISMA in multi-threshold image segmentation scenarios, we designed experiments of comparison between ISMA and GWO, SOA, SMA, MFO, POA, ESMA, and DFSMA. All the algorithms were run independently 30 times, and PSNR, SSIM, and FSIM were selected as the evaluation metrics. The best values are marked in bold.

Table 8, Table 9 and Table 10 show the PSNR, SSIM, and FSIM obtained for all the images through the algorithm, respectively. From the comparison results, we can see that the image segmentation quality becomes better as the thresholds increase, and PSNR, SSIM, and FSIM are all proportional to the number of thresholds.

As can be seen from Table 8, the PSNR obtained by image segmentation at different thresholds of ISMA achieved optimal values for all eight images, which were better than those of the other comparison algorithms. When the threshold was low (*d* = 2, 3), there was little difference in the PSNR values obtained by image segmentation using the other comparison algorithms. At high thresholding (*d* = 4, 5), SOA and GWO obtained poor PSNR values in most image segmentations, and ESMA and DFSMA obtained lower PSNR values than SMA in most image segmentations.

As can be seen in Table 9, the SSIM values obtained by ISMA achieved optimal values in the segmentation of all eight images, outperforming the other compared algorithms. At low thresholds (*d* = 2, 3), only ISMA obtained the optimal SSIM value for Milkdrop and Man. Each algorithm obtained the optimal SSIM value for the rest of the images, and only SOA still obtained the poor SSIM value for Lena and Barbara. When *d* = 4, SMA, POA, ESMA, and DFSMA obtained the optimal SSIM values for Cameraman, Butterfly, Barbara, and Man, respectively.

As can be seen in Table 10, the optimal FSIM values were obtained by ISMA in all eight image segmentation tests, which were better than those of the other comparison algorithms. When *d* = 2, 3, the other algorithms did not differ much in the case of Lena, Cameraman, Lake, and Barbara, but the FSIM values obtained for the remaining images were lower than those obtained by ISMA. When *d* = 4, all the algorithms except for SOA obtained the optimal FSIM values for Cameraman. When *d* = 5, the optimal FSIM values were obtained for Lena by all the algorithms except for SOA and POA.

To test the stability of ISMA in the image segmentation task, 30 independent runs were performed on the images in order to obtain the optimal fitness values, and the mean and variance of the optimal fitness values were selected as the evaluation indices. Table 11 and Table 12 shows the mean and std of fitness obtained by the algorithms for all the images, respectively.

It can be seen from the table that the image segmentation result of SOA was unstable in the 30 independent operations, and the values of the mean and std were the largest. The mean and std obtained by GWO were slightly better than those obtained by SOA, and the image segmentation was still not stable. When *d* = 2, 3, 4, SMA, POA, MFO, ESMA, DFSMA, and SMA obtained the same mean values for most of the images, but ISMA obtained a lower std value and was able to complete the image segmentation task stably. When *d* = 5, only ISMA obtained the optimal mean and std.

The average CPU times of the different algorithms, considering all cases, are provided in Table 13. As can be seen from the table, MFO and SOA each achieved the lowest computation time for most of the images. SMA also achieved the optimal computation time for a small number of images. ISMA performed second to SMA and better than the other residual algorithms. POA performed poorly in terms of the image segmentation time. ISMA improved the image segmentation accuracy while maintaining the runtime.

**Figure 12:**
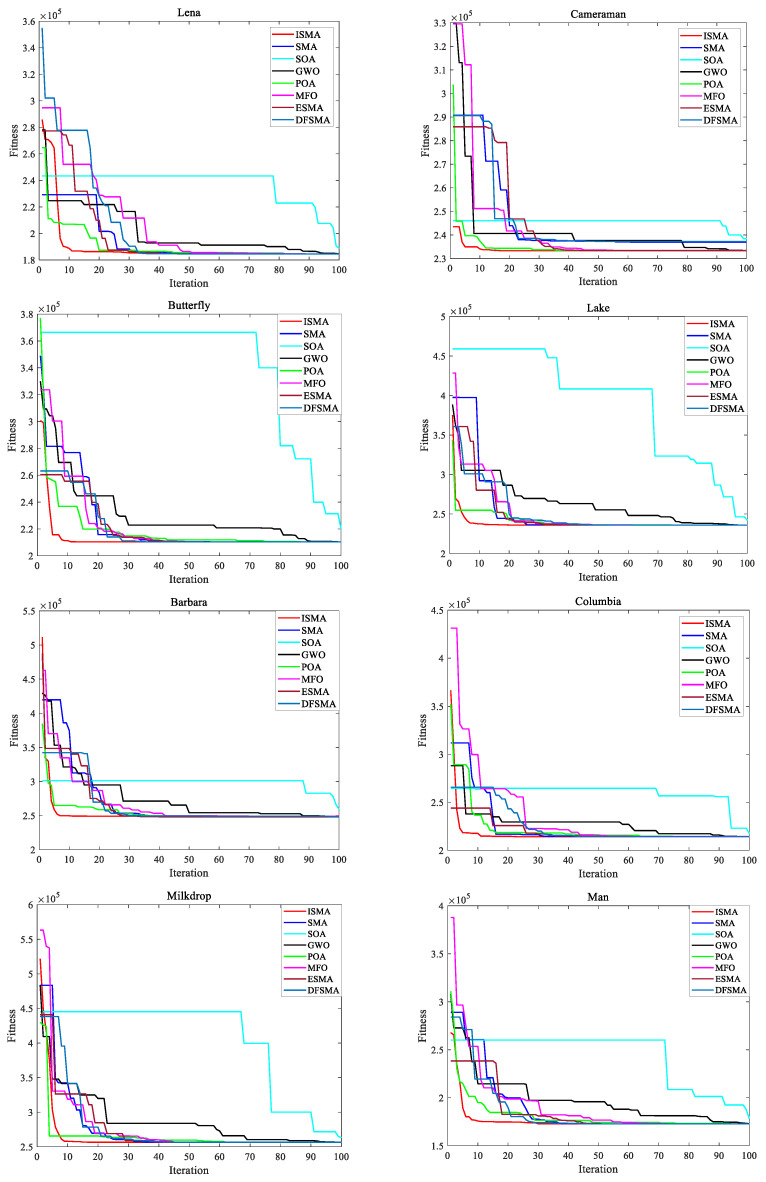
Convergence behavior of the algorithms for all images when *d* = 5.

To better reflect the convergence of the five algorithms, the five-threshold segmentation convergence curves of the eight images were plotted, as shown in Figure 12. From the figure, it can be seen that it was easy for SOA to fall into the local optimum during image segmentation, and the obtained adaptation value was poor. GWO performed slightly better than SOA for the eight images. For the eight images, all the algorithms except SOA eventually converged to the optimal fitness value. However, ISMA was the first to converge and the fastest to converge, followed by POA. This was made possible by the adaptive probability threshold used by ISMA, which allows the sticklebacks to select a predation strategy suitable for the current population, thus increasing the convergence speed of the algorithm.

In summary, ISMA can converge to the optimal solution stably, and there are some improvements in the convergence speed and segmentation accuracy compared with the other seven algorithms, and it can obtain high-quality segmented images. Therefore, this paper proposed that multi-level thresholding image segmentation based on the improved slime mould algorithm and symmetric cross-entropy can be effectively applied to image multi-threshold segmentation tasks, with an excellent performance.

## 7. Conclusions

In this paper, we introduced an improved slime mould algorithm, ISMA, for multi-threshold image segmentation tasks. The slime mould algorithm can easily fall into the local optimum, as in the case of other intelligent algorithms, and cannot solve complex real-world problems. In this work, EOBL improved the quality and diversity of the initial population to accelerate the convergence speed. The adaptive probability threshold adjusted the selection probability of the slime mould to enhance the ability of the algorithm to jump out of the local optimum, and the historical leader strategy selected the optimal historical information as the leader for the position update so as to improve the convergence accuracy.

We evaluated the optimization performance of ISMA using 14 benchmark test functions. The experimental results showed that ISMA has a better overall capability in terms of the optimization accuracy and convergence speed compared with the original SMA, as well as the other well-known MAs. Subsequently, ISMA was applied to solve the multi-threshold image segmentation task, and symmetric cross-entropy was used as the objective function to obtain the optimal threshold value. Experimental evaluation metrics such as the PSNR, SSIM, and FSIM were used to test the quality of the segmented images. The experimental results demonstrated that: (1) the image segmentation quality is better than that obtained by Otsu, Kapur’s entropy, and minimum cross-entropy when symmetric cross-entropy is taken as the objective function; and (2) ISMA achieves clearer image segmentation results compared with the other MAs. Finally, we conclude that multi-threshold image segmentation based on ISMA and symmetric cross-entropy outperforms the other selected MAs in terms of the segmentation accuracy and can better preserve the edge details of the images.

Although ISMA has achieved excellent results in benchmark function testing and image segmentation, it still has some shortcomings when solving image tasks. Future work will focus on reducing the computation time without degrading the performance of ISMA and applying multi-threshold image segmentation based on ISMA and symmetric cross-entropy to real medical images and remote sensing image testing in order to further demonstrate its performance.

## Figures and Tables

**Table 1:** Definitions of the 14 benchmark functions.

No	Name	Range	D	*f_min_*	Type
F1	Sphere	[−100, 100]	30	0	UM
F2	Schwefel 2.22	[−10, 10]	30	0	UM
F3	Schwefel 1.2	[−100, 100]	30	0	UM
F4	Schwefel 2.21	[−100, 100]	30	0	UM
F5	Rosenbrock	[−30, 30]	30	0	UM
F6	Step	[−100, 100]	30	0	UM
F7	Quartic	[−1.28, 1.28]	30	0	UM
F8	Schwefel	[−500, 500]	30	−12,569.487	MM
F9	Rastrigin	[−5.12, 5.12]	30	0	MM
F10	Ackley	[−32, 32]	30	0	MM
F11	Griewank	[−600, 600]	30	0	MM
F12	Penalized	[−50, 50]	30	0	MM
F13	Penalized 2	[−50, 50]	30	0	MM
F14	Foxholes	[−65.536, 65.536]	2	0.998004	MM

**Table 2:** Parameter settings for each algorithm.

Algorithm	Parameters
ISMA	*Z* = 0.03
SMA	*Z* = 0.03
SOA	*F_C_* = 2, *u* = 1, *v* = 1
MFO	*b* = 1, ε = 0.001, *g* ∈ [0, 30], *C* ∈ [0, 100]
GWO	*a* ∈ [2, 0]
POA	*I* = 2, *R* = 0.2
ESMA	*Z* = 0.03
DFSMA	*Z* = 0.03

**Table 3:** Mean statistical results of the algorithms based on 14 benchmark functions.

Function	ISMA	SMA	SOA	MFO	POA	GWO	ESMA	DFSMA
F1	**0.000**	1.500 × 10^−323^	8.496 × 10^−12^	1.341 × 10^3^	1.525 × 10^−103^	1.619 × 10^−28^	5.106 × 10^−297^	2.320 × 10^−292^
F2	**0.000**	6.348 × 10^−147^	1.590 × 10^−8^	3.685 × 10	6.001 × 10^−52^	9.760 × 10^−17^	3.758 × 10^−168^	9.280 × 10^−153^
F3	**0.000**	1.446 × 10^−313^	1.258 × 10^−4^	2.109 × 10^4^	3.735 × 10^−100^	7.918 × 10^−6^	2.970 × 10^−296^	5.900 × 10^−323^
F4	**0.000**	1.007 × 10^−148^	1.758 × 10^−2^	6.794 × 10	2.443 × 10^−51^	6.649 × 10^−7^	4.231 × 10^−141^	1.983 × 10^−160^
F5	**2.550**	1.207 × 10^1^	2.822 × 10	2.688 × 10^6^	2.810 × 10	2.682 × 10	5.415	3.262
F6	**4.239 × 10^−4^**	7.500 × 10^−3^	3.306	1.680 × 10^3^	2.829	7.552 × 10^−1^	5.803 × 10^−3^	5.431 × 10^−3^
F7	**1.211 × 10^−4^**	1.805 × 10^−4^	2.631 × 10^−3^	5.766	2.363 × 10^−4^	1.657 × 10^−3^	1.950 × 10^−4^	1.685 × 10^−4^
F8	**−1.257 × 10^4^**	**−1.257 × 10^4^**	−4.861 × 10^3^	9.933 × 10^2^	−7.642 × 10^3^	−5.930 × 10^3^	−1.256 × 10^4^	−1.256 × 10^4^
F9	**0.000**	**0.000**	1.297	1.714 × 10^2^	**0.000**	4.815	**0.000**	**0.000**
F10	**8.882 × 10^−16^**	**8.882 × 10^−16^**	1.996 × 10	1.448 × 10	3.257 × 10^−15^	9.456 × 10^−14^	**8.882 × 10^−16^**	**8.882 × 10^−16^**
F11	**0.000**	**0.000**	2.989 × 10^−2^	1.591 × 10	**0.000**	4.596 × 10^−3^	**0.000**	**0.000**
F12	**3.098 × 10^−4^**	5.500 × 10^−3^	3.439 × 10^−1^	8.534 × 10^6^	1.926 × 10^−1^	5.065 × 10^−2^	5.237 × 10^−3^	4.620 × 10^−3^
F13	**1.700 × 10^−3^**	1.240 × 10^−2^	2.057	2.381 × 10^2^	2.589	5.603 × 10^−1^	6.689 × 10^−3^	6.933 × 10^−3^
F14	**9.980 × 10^−1^**	1.013	2.150	1.823	1.823	4.655	9.981 × 10^−1^	9.981 × 10^−1^

**Table 4:** Std statistical results of the algorithms based on 14 benchmark functions.

Function	ISMA	SMA	SOA	MFO	POA	GWO	ESMA	DFSMA
F1	**0.000**	**0.000**	1.787 × 10^−11^	4.340 × 10^3^	7.328 × 10^−103^	2.412 × 10^−27^	**0.000**	**0.000**
F2	**0.000**	3.477 × 10^−146^	1.791 × 10^−8^	2.778 × 10	2.117 × 10^−51^	5.044 × 10^−17^	1.776 × 10^−156^	5.082 × 10^−152^
F3	**0.000**	**0.000**	5.100 × 10^−4^	9.875 × 10^3^	1.932 × 10^−99^	1.388 × 10^−5^	**0.000**	**0.000**
F4	**0.000**	5.518 × 10^−148^	7.711 × 10^−2^	6.246	1.187 × 10^−50^	5.612 × 10^−7^	2.317 × 10^−140^	1.086 × 10^−159^
F5	**5.023**	1.354 × 10^01^	2.883 × 10	2.004 × 10^2^	8.098 × 10^−1^	2.711 × 10	9.366	7.662
F6	**7.757 × 10^−4^**	3.100 × 10^−3^	4.505 × 10^−1^	3.795 × 10^3^	6.616 × 10^−1^	3.078 × 10^−1^	3.336 × 10^−3^	2.451 × 10^−3^
F7	**1.110 × 10^−4^**	1.431 × 10^−4^	2.043 × 10^−3^	1.143 × 10^1^	1.797 × 10^−04^	8.672 × 10^−4^	1.527 × 10^−4^	1.715 × 10^−4^
F8	**3.087 × 10^−1^**	1.575 × 10^1^	4.834 × 10^2^	3.985 × 10^3^	7.531 × 10^02^	8.413 × 10^2^	4.327 × 10^−1^	3.812 × 10^−1^
F9	**0.000**	**0.000**	2.348	4.479 × 10^1^	**0.000**	7.065	**0.000**	**0.000**
F10	**0.000**	**0.000**	1.540 × 10^−3^	6.998	1.703 × 10^−15^	1.410 × 10^−14^	**0.000**	**0.000**
F11	**0.000**	**0.000**	5.084 × 10^−2^	3.407 × 10^1^	**0.000**	9.000 × 10^−3^	**0.000**	**0.000**
F12	**6.516 × 10^−4^**	5.000 × 10^−3^	1.016 × 10^−1^	2.492 × 10^2^	7.090 × 10^−02^	1.918 × 10^−2^	7.366 × 10^−3^	6.237 × 10^−3^
F13	**2.100 × 10^−3^**	1.230 × 10^−2^	1.449 × 10^−1^	8.193 × 10^2^	4.345 × 10^−1^	2.567 × 10^−1^	8.739 × 10^−3^	8.789 × 10^−3^
F14	**3.593 × 10^−13^**	6.655 × 10^−2^	1.891	1.399	1.399	4.146	5.955 × 10^−13^	4.627 × 10^−13^

**Table 5:** Computation time(s) statistical results of the algorithms based on 14 benchmark functions.

Function	ISMA	SMA	SOA	MFO	POA	GWO	ESMA	DFSMA
F1	3.292 × 10^−1^	3.207 × 10^−1^	3.090 × 10^−1^	2.812 × 10^−1^	**1.963 × 10^−1^**	5.205 × 10^−1^	3.533 × 10^−1^	3.450 × 10^−1^
F2	3.442 × 10^−2^	3.424 × 10^−1^	3.541 × 10^−1^	3.331 × 10^−1^	**2.219 × 10^−1^**	5.747 × 10^−1^	3.755 × 10^−1^	3.739 × 10^−1^
F3	4.387 × 10^−1^	4.348 × 10^−1^	4.248 × 10^−1^	**3.998 × 10^−1^**	4.523 × 10^−1^	6.052 × 10^−1^	4.765 × 10^−1^	4.739 × 10^−1^
F4	3.334 × 10^−1^	3.262 × 10^−1^	2.783 × 10^−1^	2.425 × 10^−1^	**1.583 × 10^−1^**	4.419 × 10^−1^	3.593 × 10^−1^	3.354 × 10^−1^
F5	3.522 × 10^−1^	3.433 × 10^−1^	2.885 × 10^−1^	2.510 × 10^−1^	**1.822 × 10^−1^**	4.630 × 10^−1^	3.854 × 10^−1^	3.702 × 10^−1^
F6	3.388 × 10^−1^	3.290 × 10^−1^	2.886 × 10^−1^	2.597 × 10^−1^	**1.862 × 10^−1^**	4.395 × 10^−1^	3.685 × 10^−1^	3.539 × 10^−1^
F7	3.892 × 10^−1^	3.812 × 10^−1^	3.171 × 10^−1^	2.853 × 10^−1^	**2.595 × 10^−1^**	4.876 × 10^−1^	4.282 × 10^−1^	1.071 × 10^−1^
F8	3.535 × 10^−1^	3.383 × 10^−1^	3.408 × 10^−1^	2.884 × 10^−1^	**2.670 × 10^−1^**	4.838 × 10^−1^	3.938 × 10^−1^	3.638 × 10^−1^
F9	3.325 × 10^−1^	3.233 × 10^−1^	2.813 × 10^−1^	2.532 × 10^−1^	**2.013 × 10^−1^**	4.588 × 10^−1^	3.721 × 10^−1^	3.422 × 10^−1^
F10	3.396 × 10^−1^	3.255 × 10^−1^	2.830 × 10^−1^	2.560 × 10^−1^	**1.781 × 10^−1^**	4.495 × 10^−1^	3.654 × 10^−1^	3.598 × 10^−1^
F11	3.571 × 10^−1^	3.347 × 10^−1^	3.008 × 10^−1^	2.652 × 10^−1^	**2.005 × 10^−1^**	4.640 × 10^−1^	3.933 × 10^−1^	3.654 × 10^5^
F12	5.140 × 10^−1^	4.973 × 10^−1^	4.824 × 10^−1^	**4.385 × 10^−1^**	5.666 × 10^−1^	6.634 × 10^−1^	5.535 × 10^−1^	5.449 × 10^−1^
F13	5.017 × 10^−1^	4.978 × 10^−1^	4.806 × 10^−1^	**4.500 × 10^−1^**	5.501 × 10^−1^	6.495 × 10^−1^	5.727 × 10^−1^	5.220 × 10^−1^
F14	5.090 × 10^−1^	5.042 × 10^−1^	4.681 × 10^−1^	**4.547 × 10^−1^**	8.880 × 10^−1^	4.741 × 10^−1^	5.748 × 10^−1^	5.382 × 10^−1^

**Table 6:** The PSNR, SSIM, and FSIM values obtained by segmentation criterion.

Images	*d*	Symmetric Cross-Entropy			Minimum Cross-Entropy			Otsu			Kapur’s Entropy		
		PSNR	SSIM	FSIM	PSNR	SSIM	FSIM	PSNR	SSIM	FSIM	PSNR	SSIM	FSIM
**Lena**	2	**13.2058**	**0.4969**	**0.6978**	12.2497	0.4949	0.6976	12.0066	0.4723	0.6897	7.7212	0.1290	0.5866
	3	**15.7899**	**0.5628**	**0.7660**	15.6702	0.5607	0.7654	15.6612	0.5362	0.7542	13.3143	0.5223	0.6812
	4	**16.5512**	**0.5776**	**0.8029**	16.2183	0.5632	0.7956	16.2183	0.5580	0.7954	15.4882	0.5661	0.6985
	5	**17.0899**	**0.6802**	**0.8305**	16.7296	0.6115	0.8305	16.9276	0.5814	0.8289	17.0600	0.5998	0.7196
**Cameraman**	2	**12.0475**	0.5555	**0.7662**	11.5227	**0.5562**	0.7554	11.5288	0.5551	0.7549	11.3573	0.4941	0.6504
	3	**12.8391**	**0.5982**	**0.8097**	11.5551	0.5980	0.8074	11.5670	0.5739	0.7910	12.4609	0.5567	0.6530
	4	**16.1448**	**0.6089**	**0.8344**	12.7883	0.6089	0.8344	12.7883	0.5984	0.8192	12.9698	0.5646	0.6742
	5	**16.6072**	**0.6384**	**0.8584**	14.8859	0.6276	0.8447	15.7175	0.6175	0.8576	16.4663	0.5876	0.6746
**Butterfly**	2	**13.2788**	**0.5266**	**0.7363**	13.1412	0.5266	0.7330	13.1412	0.4730	0.7363	11.7625	0.3130	0.7101
	3	**15.5515**	**0.5759**	**0.7914**	14.9690	0.5759	0.7338	14.9690	0.5603	0.7738	14.0402	0.4095	0.7524
	4	**16.4086**	**0.6147**	**0.8153**	15.9752	0.6147	0.8079	16.0354	0.6183	0.8153	16.0276	0.6435	0.7821
	5	**16.6381**	**0.6474**	**0.8281**	16.0354	0.6327	0.8143	16.1095	0.7103	0.8182	16.5343	0.6478	0.8136
**Lake**	2	**13.1104**	**0.5017**	**0.7314**	12.9005	0.5002	0.7313	12.9179	0.4781	0.7313	13.3854	0.4398	0.6234
	3	**16.1352**	**0.5589**	**0.7908**	14.0479	0.5589	0.7835	14.0479	0.5180	0.7835	16.2402	0.5150	0.6565
	4	**18.0386**	**0.6572**	**0.8391**	18.0386	0.6071	0.8257	17.3537	0.5589	0.8257	15.8882	0.5346	0.6918
	5	**18.6824**	**0.6948**	**0.8623**	18.4376	0.6542	0.8412	18.2994	0.6071	0.8360	15.8486	0.6575	0.7275
**Barbara**	**2**	**14.7227**	**0.4756**	**0.7278**	12.6622	0.4756	0.7278	13.1800	0.4631	0.7261	14.7227	0.4663	0.6874
	3	**16.3754**	**0.5432**	**0.7943**	16.3754	0.5432	0.7942	15.6470	0.5347	0.7943	15.8715	0.4933	0.7339
	4	**16.9175**	**0.6023**	**0.8304**	16.8855	0.6023	0.8301	16.6012	0.5770	0.8104	15.9885	0.5687	0.8294
	5	**17.9234**	**0.6553**	**0.8512**	17.0108	0.6545	0.8500	17.1495	0.6064	0.8289	17.9234	0.5913	0.8441
**Columbia**	2	**13.8785**	**0.3900**	**0.7056**	11.2266	0.3900	0.6707	12.7799	0.3157	0.6707	13.8758	0.2524	0.7020
	3	**15.3874**	**0.5372**	**0.7812**	13.0064	0.5372	0.7338	13.0064	0.4605	0.7618	15.3316	0.3551	0.7812
	4	**16.2809**	**0.6030**	**0.8030**	14.7853	0.5993	0.7827	15.2117	0.6014	0.8000	15.9069	0.3952	0.8030
	5	**16.7908**	**0.6269**	**0.8200**	15.2117	0.6250	0.8086	15.9069	0.6250	0.8042	16.0853	0.3905	0.8042
**Milkdrop**	2	**15.8750**	**0.6458**	**0.7313**	12.9792	0.5851	0.7242	**15.8750**	0.5552	0.7266	13.3613	0.5906	0.7075
	3	**18.4471**	**0.6644**	**0.7845**	15.4290	0.6063	0.7511	17.4501	0.5968	0.7544	18.4471	0.5956	0.7371
	4	**19.3641**	**0.6849**	**0.8207**	17.2172	0.6739	0.7627	18.3448	0.6285	0.7985	19.2545	0.6035	0.7525
	5	**19.7948**	**0.6880**	**0.8309**	19.2545	0.6849	0.8303	19.3641	0.6621	0.8262	19.3641	0.6727	0.7544
**Man**	2	**14.6403**	**0.4300**	**0.6925**	11.0410	0.3867	0.6845	12.2389	0.3681	0.6845	14.6403	0.4033	0.6144
	3	**16.4136**	**0.4763**	**0.7664**	13.7597	0.4708	0.7657	14.1385	0.4634	0.7636	16.4136	0.4313	0.6393
	4	**17.3328**	**0.5114**	**0.8147**	13.9758	0.5114	0.7959	16.5070	0.5053	0.7959	16.5070	0.4696	0.6393
	5	**17.7032**	**0.5753**	**0.8453**	16.1596	0.5570	0.8362	17.3328	0.5517	0.8362	**17.7032**	0.4696	0.6545

**Table 7:** The best thresholds obtained by segmentation criterion.

Images	*d*	Symmetric Cross-Entropy	Minimum Cross-Entropy	Otsu	Kapur’s Entropy
**Lena**	2	82 140	82 142	92 151	163 220
	3	73 120 166	74 121 167	80 126 170	59 164 220
	4	71 109 140 175	70 109 139 175	74 113 145 180	57 60 164 221
	5	62 88 118 147 180	62 88 117 146 180	73 109 136 160 188	58 162 180 217 236
**Cameraman**	2	54 137	52 137	70 144	19 193
	3	31 94 144	30 84 145	57 116 154	18 21 194
	4	30 77 124 157	29 76 125 157	40 93 140 170	1 17 20 193
	5	28 71 112 144 172	28 71 113 145 172	36 83 122 149 173	1 16 19 21 194
**Butterfly**	2	76 138	76 138	85 148	114 206
	3	67 108 158	67 107 159	75 120 170	97 125 207
	4	62 92 128 172	62 93 128 172	66 99 135 177	57 102 126 208
	5	59 82 107 137 177	57 81 104 135 176	36 83 122 149 173	57 101 126 205 235
**Lake**	2	74 143	75 142	86 155	73 228
	3	65 107 163	64 162 107	80 141 194	62 86 228
	4	60 93 145 196	60 94 145 195	68 111 158 199	9 62 86 228
	5	53 77 112 155 197	55 80 116 160 199	60 91 128 166 200	10 29 72 89 228
**Barbara**	2	74 138	74 139	82 147	54 174
	3	67 119 170	68 119 171	75 127 176	55 169 222
	4	56 93 132 176	56 92 133 177	66 106 142 182	54 128 174 223
	5	47 76 108 141 181	48 76 108 142 180	57 88 118 148 184	54 129 174 218 241
**Columbia**	2	59 110	60 109	75 130	93 177
	3	50 83 130	50 83 129	61 102 152	77 147 211
	4	45 71 105 148	45 71 104 148	50 79 115 159	74 102 162 218
	5	39 59 81 111 151	40 60 82 113 155	48 74 103 135 171	73 101 152 190 234
**Milkdrop**	2	65 140	68 142	76 154	120 173
	3	35 83 150	33 81 145	72 127 188	16 120 173
	4	33 68 99 154	33 80 127 185	51 90 132 190	1 16 121 173
	5	33 68 96 131 187	31 67 95 132 187	37 70 97 134 191	1 16 118 154 241
**Man**	2	75 130	76 130	87 142	54 181
	3	67 109 152	66 108 152	71 114 156	55 176 224
	4	60 90 122 158	60 91 122 158	68 107 141 173	50 59 176 224
	5	59 85 113 143 173	59 85 113 142 173	63 94 123 151 182	1 50 58 176 225

**Table 8:** The PSNR values obtained by algorithm for all images.

Images	*d*	ISMA	GWO	SOA	SMA	POA	MFO	ESMA	DFSMA
**Lena**	2	**13.2058**	**13.2058**	**13.2058**	**13.2058**	12.0066	12.0066	12.0066	12.0066
	3	**15.7899**	15.7038	15.5352	15.7810	15.5612	15.5612	15.6512	15.6512
	4	**16.5512**	16.4458	16.4631	16.5291	16.2183	16.2183	16.2183	16.2183
	5	**17.0899**	16.7296	16.7296	16.7457	16.6878	16.7296	16.7296	16.7296
**Cameraman**	2	**12.0475**	11.9735	11.9735	**12.0475**	11.5288	11.5288	11.5288	11.5288
	3	**12.8391**	12.7378	12.6314	12.7460	11.5670	11.5670	11.5670	11.5670
	4	**16.1448**	16.1086	12.8239	16.1432	12.7883	12.7883	12.7883	12.7883
	5	**16.6072**	16.3075	13.2107	16.4663	15.7326	15.7175	14.7133	15.4820
**Butterfly**	2	**13.2788**	13.2280	13.1618	13.2280	13.1412	13.1412	13.1412	13.1412
	3	**15.5515**	15.5453	15.5163	15.4508	14.9690	14.9690	14.9690	14.9690
	4	**16.4086**	16.2999	16.2608	16.3081	16.0354	16.0354	15.9752	16.0354
	5	**16.6381**	16.3552	16.3139	16.4275	16.1095	16.1095	16.0354	16.1095
**Lake**	2	**13.1104**	**13.1104**	13.0815	**13.1104**	12.9179	12.9179	**12.9179**	**12.9179**
	3	**16.1352**	16.0206	15.9760	16.0328	14.0479	14.0249	14.0479	14.0479
	4	**18.0386**	**18.0386**	15.1716	**18.0386**	**18.0386**	**18.0386**	**18.0386**	**18.0386**
	5	**18.6824**	18.3881	18.1749	18.4822	18.3551	18.4108	18.4822	18.4822
**Barbara**	**2**	**14.7227**	12.6622	12.6622	12.6622	12.6622	12.6622	12.6622	12.6622
	**3**	**16.3754**	16.3754	16.2787	16.3754	16.3754	16.3754	16.3754	16.3754
	**4**	**16.9175**	16.8855	16.8855	16.8855	16.8855	16.8855	16.8855	16.8855
	**5**	**17.9234**	17.0108	17.3833	17.0108	17.1300	17.1063	17.0108	17.0108
**Columbia**	**2**	**13.8785**	11.2266	11.2266	11.2266	11.2266	11.2266	11.2266	11.2266
	**3**	**15.3874**	13.0064	13.0064	13.0064	13.0064	13.0644	13.0644	13.0064
	**4**	**16.2809**	14.7119	14.7523	14.7119	14.7119	14.7119	14.7119	14.7119
	**5**	**16.7908**	14.2645	14.2819	14.5631	14.2645	14.2645	14.5631	15.2117
**Milkdrop**	**2**	**15.875**	13.3168	13.4058	13.3168	13.3168	13.3168	13.3168	13.3168
	**3**	**18.4471**	15.3029	15.3029	15.3029	15.3029	15.3029	15.3029	15.3029
	**4**	**19.3641**	17.2142	17.2423	17.2142	17.2142	17.2142	17.2142	17.2142
	**5**	**19.7948**	19.2476	19.2335	19.2476	19.2476	19.2476	19.2476	19.2476
**Man**	**2**	**14.6403**	11.0410	11.2319	11.0410	11.0410	11.0410	11.0410	11.0410
	**3**	**16.4136**	13.6038	13.0089	13.6038	13.6038	13.6038	13.6038	13.6038
	**4**	**17.3328**	14.4373	14.4710	13.9758	13.9758	13.9758	13.9758	13.9758
	**5**	**17.7032**	16.0751	16.6173	16.1596	16.1596	16.1286	16.1596	16.1596

**Table 9:** The SSIM values obtained by algorithm for all images.

Images	*d*	ISMA	GWO	SOA	SMA	POA	MFO	ESMA	DFSMA
**Lena**	2	**0.4969**	**0.4969**	**0.4969**	**0.4969**	**0.4969**	**0.4969**	**0.4969**	**0.4969**
	3	**0.5628**	**0.5628**	0.5626	**0.5628**	**0.5628**	**0.5628**	**0.5628**	**0.5628**
	4	**0.5776**	0.5632	0.5632	0.5632	0.5632	0.5632	0.5632	0.5632
	5	**0.6802**	0.6115	0.6115	0.6129	0.6090	0.6115	0.6115	0.6115
**Cameraman**	2	**0.5555**	**0.5555**	**0.5555**	**0.5555**	**0.5555**	**0.5555**	**0.5555**	**0.5555**
	3	**0.5982**	**0.5982**	**0.5982**	**0.5982**	**0.5982**	**0.5982**	**0.5982**	**0.5982**
	4	**0.6089**	**0.6089**	0.6080	**0.6089**	**0.6089**	**0.6089**	**0.6089**	**0.6089**
	5	**0.6384**	0.6168	0.6159	0.6357	0.6347	0.6357	0.6222	0.6357
**Butterfly**	2	**0.5266**	**0.5266**	**0.5266**	**0.5266**	**0.5266**	**0.5266**	**0.5266**	**0.5266**
	3	**0.5759**	**0.5759**	**0.5759**	**0.5759**	**0.5759**	**0.5759**	**0.5759**	**0.5759**
	4	**0.6147**	**0.6147**	0.6039	**0.6147**	**0.6147**	**0.6147**	**0.6147**	**0.6147**
	5	**0.6474**	0.6308	0.6165	0.6313	0.6303	0.6303	0.6327	0.6303
**Lake**	2	**0.5017**	**0.5017**	**0.5017**	**0.5017**	**0.5017**	**0.5017**	**0.5017**	**0.5017**
	3	**0.5635**	0.5589	0.5585	0.5589	0.5589	**0.5635**	0.5589	0.5589
	4	**0.6582**	0.6071	0.5905	0.6071	0.6071	0.6071	0.6071	0.6071
	5	**0.6948**	0.6582	0.5995	0.6582	0.6575	0.6631	0.6607	0.6607
**Barbara**	**2**	**0.4756**	**0.4756**	**0.4756**	**0.4756**	**0.4756**	**0.4756**	**0.4756**	**0.4756**
	**3**	**0.5432**	**0.5432**	0.5413	**0.5432**	**0.5432**	**0.5432**	**0.5432**	**0.5432**
	**4**	**0.6023**	**0.6023**	0.5735	**0.6023**	**0.6023**	**0.6023**	**0.6023**	**0.6023**
	**5**	**0.6553**	0.6545	0.6003	0.6545	**0.6553**	0.6477	0.6545	0.6545
**Columbia**	**2**	**0.3900**	**0.3900**	**0.3900**	**0.3900**	**0.3900**	**0.3900**	**0.3900**	**0.3900**
	**3**	**0.5372**	**0.5372**	**0.5372**	**0.5372**	**0.5372**	**0.5372**	**0.5372**	**0.5372**
	**4**	**0.6030**	0.5993	0.5946	0.5993	0.5993	0.5993	0.5993	0.5993
	**5**	**0.6269**	0.6239	0.6298	0.6195	0.6239	0.6239	0.6195	0.6250
**Milkdrop**	**2**	**0.6458**	0.5956	0.5956	0.5956	0.5969	0.5956	0.5956	0.5964
	**3**	**0.6644**	0.6035	0.6050	0.6035	0.6035	0.6035	0.6035	0.6035
	**4**	**0.6849**	0.6727	0.6712	0.6727	0.6727	0.6727	0.6727	0.6727
	**5**	**0.6880**	0.6813	0.6836	0.6813	0.6813	0.6813	0.6813	0.6813
**Man**	**2**	**0.4300**	0.3867	0.3878	0.3867	0.3867	0.3867	0.3867	0.3867
	**3**	**0.4763**	0.4708	0.4708	0.4708	0.4708	0.4708	0.4708	0.4708
	**4**	**0.5114**	0.5064	0.5079	**0.5114**	**0.5114**	**0.5114**	**0.5114**	**0.5114**
	**5**	**0.5753**	0.5530	0.5723	0.5570	0.5570	0.5639	0.5570	0.5570

**Table 10:** The FSIM values obtained by algorithm for all images.

Images	*d*	ISMA	GWO	SOA	SMA	POA	MFO	ESMA	DFSMA
**Lena**	2	**0.6978**	**0.6978**	**0.6978**	**0.6978**	**0.6978**	**0.6978**	**0.6978**	**0.6978**
	3	**0.7660**	**0.7660**	0.7658	**0.7660**	**0.7660**	**0.7660**	**0.7660**	**0.7660**
	4	**0.8029**	0.8006	0.7923	0.8015	0.7954	0.7654	0.7954	0.7954
	5	**0.8305**	**0.8305**	0.8196	**0.8305**	0.8297	**0.8305**	**0.8305**	**0.8305**
**Cameraman**	2	**0.7662**	**0.7628**	**0.7628**	**0.7628**	0.7549	0.7549	0.7549	0.7549
	3	**0.8097**	**0.8097**	**0.8097**	**0.8097**	**0.8097**	**0.8097**	**0.8097**	**0.8097**
	4	**0.8344**	**0.8344**	0.8338	**0.8344**	**0.8344**	**0.8344**	**0.8344**	**0.8344**
	5	**0.8584**	0.8479	0.8405	0.8580	**0.8584**	0.8580	0.8419	0.8577
**Butterfly**	2	**0.7363**	0.7357	0.7357	**0.7363**	0.7330	0.7330	0.7330	0.7330
	3	**0.7914**	0.7900	0.7893	0.7909	0.7738	0.7738	0.7738	0.7738
	4	**0.8153**	0.8134	0.8130	0.8139	0.8079	0.8079	0.8079	0.8079
	5	**0.8281**	0.8260	0.8238	0.8275	0.8182	0.8182	0.8143	0.8182
**Lake**	2	**0.7314**	**0.7314**	**0.7314**	**0.7314**	**0.7314**	**0.7314**	**0.7314**	**0.7314**
	3	**0.7908**	0.7889	0.7887	0.7889	0.7835	0.7818	0.7835	0.7835
	4	**0.8391**	0.8361	0.8043	0.8361	0.8257	0.8257	0.8257	0.8257
	5	**0.8623**	0.8617	0.8271	0.8617	0.8348	0.8329	0.8411	0.8411
**Barbara**	**2**	**0.7278**	**0.7278**	**0.7278**	**0.7278**	**0.7278**	**0.7278**	**0.7278**	**0.7278**
	**3**	**0.7943**	0.7942	0.7933	0.7942	0.7942	0.7942	0.7942	0.7942
	**4**	**0.8304**	0.8301	**0.8304**	0.8301	0.8301	0.8301	0.8301	0.8301
	**5**	**0.8512**	0.8500	0.8455	0.8500	0.8511	**0.8512**	0.8500	0.8500
**Columbia**	**2**	**0.7056**	0.6707	0.6707	0.6707	0.6707	0.6707	0.6707	0.6707
	**3**	**0.7812**	0.7338	0.7338	0.7338	0.7338	0.7338	0.7338	0.7338
	**4**	**0.8030**	0.7824	0.7827	0.7824	0.7824	0.7824	0.7824	0.7824
	**5**	**0.8200**	0.7994	0.8064	0.8022	0.7994	0.7994	0.8022	0.8068
**Milkdrop**	**2**	**0.7313**	0.7223	0.7223	0.7223	0.7223	0.7223	0.7223	0.7223
	**3**	**0.7845**	0.7544	0.7576	0.7544	0.7544	0.7544	0.7544	0.7544
	**4**	**0.8207**	0.8202	0.8192	0.8202	0.8202	0.8202	0.8202	0.8202
	**5**	**0.8309**	0.8306	0.8289	0.8306	0.8306	0.8306	0.8306	0.8306
**Man**	**2**	**0.6925**	0.6845	0.6860	0.6845	0.6845	0.6845	0.6845	0.6845
	**3**	**0.7664**	0.7636	0.7584	0.7636	0.7636	0.7636	0.7636	0.7636
	**4**	**0.8147**	0.7967	0.7968	0.7959	0.7959	0.7959	0.7959	0.7959
	**5**	**0.8453**	0.8349	0.8386	0.8362	0.8362	0.8369	0.8362	0.8362

**Table 11:** The mean of fitness obtained by the algorithms for all the images.

Images	*d*	ISMA	GWO	SOA	SMA	POA	MFO	ESMA	DFSMA
**Lena**	2	**7.336 × 10^5^**	**7.336 × 10^5^**	**7.336 × 10^5^**	**7.336 × 10^5^**	**7.336 × 10^5^**	**7.336 × 10^5^**	**7.336 × 10^5^**	**7.336 × 10^5^**
	3	**3.929 × 10^5^**	3.931 × 10^5^	4.012 × 10^5^	**3.929 × 10^5^**	**3.929 × 10^5^**	**3.929 × 10^5^**	**3.929 × 10^5^**	**3.929 × 10^5^**
	4	**2.610 × 10^5^**	2.616 × 10^5^	3.305 × 10^5^	**2.610 × 10^5^**	**2.610 × 10^5^**	**2.610 × 10^5^**	**2.610 × 10^5^**	**2.610 × 10^5^**
	5	**1.845 × 10^5^**	1.855 × 10^5^	2.820 × 10^5^	**1.845 × 10^5^**	**1.845 × 10^5^**	1.855 × 10^5^	1.855 × 10^5^	1.855 × 10^5^
**Cameraman**	2	**7.909 × 10^5^**	**7.909 × 10^5^**	**7.909 × 10^5^**	**7.909 × 10^5^**	**7.909 × 10^5^**	**7.909 × 10^5^**	**7.909 × 10^5^**	**7.909 × 10^5^**
	3	**4.159 × 10^5^**	4.163 × 10^5^	4.161 × 10^5^	**4.159 × 10^5^**	**4.159 × 10^5^**	**4.159 × 10^5^**	**4.159 × 10^5^**	**4.159 × 10^5^**
	4	**3.027 × 10^5^**	3.030 × 10^5^	3.133 × 10^5^	**3.027 × 10^5^**	**3.027 × 10^5^**	**3.027 × 10^5^**	**3.027 × 10^5^**	**3.027 × 10^5^**
	5	**2.334 × 10^5^**	2.353 × 10^5^	2.795 × 10^5^	2.352 × 10^5^	**2.334 × 10^5^**	2.351 × 10^5^	2.348 × 10^5^	2.337 × 10^5^
**Butterfly**	2	**7.835 × 10^5^**	**7.835 × 10^5^**	**7.835 × 10^5^**	**7.835 × 10^5^**	**7.835 × 10^5^**	**7.835 × 10^5^**	**7.835 × 10^5^**	**7.835 × 10^5^**
	3	**4.570 × 10^5^**	**4.570 × 10^5^**	4.609 × 10^5^	**4.570 × 10^5^**	**4.570 × 10^5^**	**4.570 × 10^5^**	**4.570 × 10^5^**	**4.570 × 10^5^**
	4	**2.864 × 10^5^**	2.868 × 10^5^	3.655 × 10^5^	**2.864 × 10^5^**	**2.864 × 10^5^**	**2.864 × 10^5^**	**2.864 × 10^5^**	**2.864 × 10^5^**
	5	**2.104 × 10^5^**	2.111 × 10^5^	3.059 × 10^5^	**2.104 × 10^5^**	**2.104 × 10^5^**	**2.104 × 10^5^**	**2.104 × 10^5^**	**2.104 × 10^5^**
**Lake**	2	**7.488 × 10^5^**	**7.488 × 10^5^**	**7.488 × 10^5^**	**7.488 × 10^5^**	**7.488 × 10^5^**	**7.488 × 10^5^**	**7.488 × 10^5^**	**7.488 × 10^5^**
	3	**4.954 × 10^5^**	4.956 × 10^5^	4.985 × 10^5^	**4.954 × 10^5^**	**4.954 × 10^5^**	**4.954 × 10^5^**	**4.954 × 10^5^**	**4.954 × 10^5^**
	4	**3.311 × 10^5^**	3.327 × 10^5^	3.785 × 10^5^	**3.311 × 10^5^**	**3.311 × 10^5^**	**3.311 × 10^5^**	**3.311 × 10^5^**	**3.311 × 10^5^**
	5	**2.359 × 10^5^**	2.366 × 10^5^	3.184 × 10^5^	2.360 × 10^5^	**2.359 × 10^5^**	2.360 × 10^5^	2.361 × 10^5^	2.360 × 10^5^
**Barbara**	**2**	**8.959 × 10^5^**	**8.959 × 10^5^**	8.966 × 10^5^	**8.959 × 10^5^**	**8.959 × 10^5^**	**8.959 × 10^5^**	**8.959 × 10^5^**	**8.959 × 10^5^**
	**3**	**5.551 × 10^5^**	5.552 × 10^5^	5.578 × 10^5^	**5.551 × 10^5^**	**5.551 × 10^5^**	**5.551 × 10^5^**	**5.551 × 10^5^**	**5.551 × 10^5^**
	**4**	**3.583 × 10^5^**	3.585 × 10^5^	3.629 × 10^5^	**3.583 × 10^5^**	**3.583 × 10^5^**	**3.583 × 10^5^**	**3.583 × 10^5^**	**3.583 × 10^5^**
	**5**	**2.482 × 10^5^**	2.493 × 10^5^	2.597 × 10^5^	**2.482 × 10^5^**	**2.482 × 10^5^**	**2.482 × 10^5^**	2.483 × 10^5^	2.483 × 10^5^
**Columbia**	**2**	**7.898 × 10^5^**	**7.898 × 10^5^**	7.899 × 10^5^	**7.898 × 10^5^**	**7.898 × 10^5^**	**7.898 × 10^5^**	**7.898 × 10^5^**	**7.898 × 10^5^**
	**3**	**4.692 × 10^5^**	**4.692 × 10^5^**	4.710 × 10^5^	**4.692 × 10^5^**	**4.692 × 10^5^**	**4.692 × 10^5^**	**4.692 × 10^5^**	**4.692 × 10^5^**
	**4**	**3.038 × 10^5^**	**3.038 × 10^5^**	3.120 × 10^5^	**3.038 × 10^5^**	**3.038 × 10^5^**	**3.038 × 10^5^**	**3.038 × 10^5^**	**3.038 × 10^5^**
	**5**	**2.143 × 10^5^**	2.152 × 10^5^	2.329 × 10^5^	2.145 × 10^5^	2.143 × 10^5^	2.148 × 10^5^	2.145 × 10^5^	2.145 × 10^5^
**Milkdrop**	**2**	**1.304 × 10^6^**	**1.304 × 10^6^**	1.305 × 10^6^	**1.304 × 10^6^**	**1.304 × 10^6^**	**1.304 × 10^6^**	**1.304 × 10^6^**	**1.304 × 10^6^**
	**3**	**6.956 × 10^5^**	**6.956 × 10^5^**	6.968 × 10^5^	**6.956 × 10^5^**	**6.956 × 10^5^**	**6.956 × 10^5^**	**6.956 × 10^5^**	**6.956 × 10^5^**
	**4**	**4.493 × 10^5^**	4.505 × 10^5^	4.548 × 10^5^	4.500 × 10^5^	4.497 × 10^5^	4.499 × 10^5^	4.506 × 10^5^	4.497 × 10^5^
	**5**	**2.561 × 10^5^**	2.577 × 10^5^	2.708 × 10^5^	2.566 × 10^5^	2.566 × 10^5^	2.566 × 10^5^	2.566 × 10^5^	2.566 × 10^5^
**Man**	**2**	**6.685 × 10^5^**	**6.685 × 10^5^**	6.687 × 10^5^	**6.685 × 10^5^**	**6.685 × 10^5^**	**6.685 × 10^5^**	**6.685 × 10^5^**	**6.685 × 10^5^**
	**3**	**3.673 × 10^5^**	3.675 × 10^5^	3.689 × 10^5^	**3.673 × 10^5^**	**3.673 × 10^5^**	**3.673 × 10^5^**	**3.673 × 10^5^**	**3.673 × 10^5^**
	**4**	**2.496 × 10^5^**	2.505 × 10^5^	2.587 × 10^5^	**2.496 × 10^5^**	**2.496 × 10^5^**	2.501 × 10^5^	**2.496 × 10^5^**	**2.496 × 10^5^**
	**5**	**1.729 × 10^5^**	1.743 × 10^5^	1.924 × 10^5^	1.730 × 10^5^	1.730 × 10^5^	1.735 × 10^5^	1.730 × 10^5^	1.731 × 10^5^

**Table 12:** The std of fitness obtained by the algorithms for all images.

Images	*d*	ISMA	GWO	SOA	SMA	POA	MFO	× 10SMA	DFSMA
**Lena**	2	**1.227 × 10^−10^**	6.904 × 10	2.368 × 10^−10^	2.368 × 10^−10^	2.368 × 10^−10^	2.368 × 10^−10^	2.368 × 10^−10^	**1.227 × 10^−10^**
	3	**0.000**	9.720 × 10^2^	2.949 × 10^4^	2.960 × 10^−10^	2.960 × 10^−10^	2.960 × 10^−10^	**0.000**	**0.000**
	4	**3.068 × 10^−11^**	1.644 × 10^3^	4.273 × 10^4^	1.184 × 10^−10^	1.184 × 10^−10^	1.184 × 10^−10^	1.184 × 10^−10^	1.184 × 10^−10^
	5	**3.068 × 10^−11^**	2.522 × 10^3^	2.942 × 10^4^	8.880 × 10^−11^	8.771 × 10	9.163 × 10	**3.068 × 10^−11^**	**3.068 × 10^−11^**
**Cameraman**	2	**1.184 × 10^−10^**	**1.184 × 10^−10^**	**1.184 × 10^−10^**	**1.184 × 10^−10^**	1.184 × 10^−10^	**1.184 × 10^−10^**	1.227 × 10^−10^	1.227 × 10^−10^
	3	**6.136 × 10^−11^**	2.216 × 10^3^	2.472 × 10^2^	1.776 × 10^−10^	1.776 × 10^−10^	1.776 × 10^−10^	**6.136 × 10^−11^**	**6.136 × 10^−11^**
	4	**0.000**	9.009 × 10^2^	1.732 × 10^4^	2.368 × 10^−10^	2.368 × 10^−10^	2.368 × 10^−10^	**0.000**	**0.000**
	5	**1.778 × 10^2^**	2.201 × 10^3^	2.626 × 10^4^	1.920 × 10^3^	1.892 × 10^3^	1.935 × 10^3^	1.875 × 10^3^	1.232 × 10^3^
**Butterfly**	2	**1.227 × 10^−10^**	2.230 × 10^2^	1.323 × 10^2^	3.552 × 10^−10^	3.552 × 10^−10^	3.552 × 10^−10^	**1.227 × 10^−10^**	**1.227 × 10^−10^**
	3	**6.136 × 10^−11^**	2.518 × 10^2^	1.034 × 10^4^	1.206 × 10	1.776 × 10^−10^	1.400 × 10	**6.136 × 10^−11^**	**6.136 × 10^−11^**
	4	**6.136 × 10^−11^**	1.030 × 10^3^	5.854 × 10^4^	1.184 × 10^−10^	1.184 × 10^−10^	1.184 × 10^−10^	**6.136 × 10^−11^**	**6.136 × 10^−11^**
	5	**4.658 × 10**	2.343 × 10^3^	4.496 × 10^4^	6.780 × 10	4.936 × 10	1.159 × 10^2^	8.012 × 10	5.252 × 10
**Lake**	2	**0.000**	1.377 × 10^2^	9.821	3.552 × 10^−10^	3.552 × 10^−10^	3.552 × 10^−10^	3.552 × 10^−10^	0.000
	3	**1.227 × 10^−10^**	1.119 × 10^3^	9.698 × 10^3^	1.776 × 10^−10^	1.776 × 10^−10^	1.776 × 10^−10^	**1.227 × 10^−10^**	**1.227 × 10^−10^**
	4	**0.000**	5.207 × 10^3^	4.114 × 10^4^	2.960 × 10^−10^	2.960 × 10^−10^	2.960 × 10^−10^	**0.000**	**0.000**
	5	**1.121 × 10^2^**	2.058 × 10^3^	4.480 × 10^4^	1.181 × 10^2^	4.672 × 10	1.192 × 10^2^	9.631 × 10	1.125 × 10^2^
**Barbara**	**2**	**0.000**	1.184 × 10^−10^	5.490 × 10^2^	1.184 × 10^−10^	1.184 × 10^−10^	1.184 × 10^−10^	**0.000**	**0.000**
	**3**	**0.000**	6.443 × 10^2^	1.810 × 10^3^	**0.000**	**0.000**	**0.000**	**0.000**	**0.000**
	**4**	**0.000**	1.028 × 10^3^	3.237 × 10^3^	**0.000**	**0.000**	**0.000**	6.136 × 10^−11^	6.136 × 10^−11^
	**5**	**1.214 × 10^2^**	2.636 × 10^3^	1.967 × 10^4^	1.710 × 10^2^	2.856 × 10^2^	2.376 × 10^2^	3.143 × 10^2^	2.982 × 10^2^
**Columbia**	**2**	**1.184 × 10^−10^**	**1.184 × 10^−10^**	2.308 × 10^2^	**1.184 × 10^−10^**	**1.184 × 10^−10^**	**1.184 × 10^−10^**	1.227 × 10^−10^	1.227 × 10^−10^
	**3**	**0.000**	1.956 × 10^2^	9.040 × 10^2^	2.960 × 10^−10^	2.960 × 10^−10^	2.960 × 10^−10^	**0.000**	**0.000**
	**4**	**0.000**	2.857 × 10^2^	3.020 × 10^4^	2.368 × 10^−10^	3.086 × 10	3.086 × 10	0.000	0.000
	**5**	**4.182 × 10**	2.346 × 10^3^	3.366 × 10^4^	4.204 × 10^2^	4.182 × 10	6.908 × 10^2^	4.372 × 10^2^	4.372 × 10^2^
**Milkdrop**	**2**	**0.000**	**0.000**	6.849 × 10^2^	**0.000**	**0.000**	**0.000**	**0.000**	**0.000**
	**3**	**0.000**	1.458 × 10^2^	6.801 × 10^2^	**0.000**	**0.000**	**0.000**	1.227 × 10^−10^	1.227 × 10^−10^
	**4**	**4.747 × 10**	3.207 × 10^3^	3.095 × 10^3^	1.567 × 10^3^	1.261 × 10^3^	1.429 × 10^3^	1.997 × 10^3^	1.307 × 10^3^
	**5**	**0.000**	4.512 × 10^3^	3.620 × 10^4^	2.960 × 10^−11^	2.810	2.960 × 10^−11^	**0.000**	**0.000**
**Man**	**2**	**1.184 × 10^−10^**	**1.184 × 10^−10^**	3.175 × 10^2^	**1.184 × 10^−10^**	**1.184 × 10^−10^**	**1.184 × 10^−10^**	1.227 × 10^−10^	1.227 × 10^−10^
	**3**	**0.000**	4.403 × 10^2^	2.117 × 10^3^	6.882	7.798	7.798	7.132	6.136 × 10^−11^
	**4**	**6.136 × 10^−11^**	2.104 × 10^3^	2.069 × 10^4^	8.880 × 10^−11^	8.880 × 10^−11^	1.128 × 10^3^	6.136 × 10^−11^	6.136 × 10^−11^
	**5**	**0.000**	3.379 × 10^3^	2.601 × 10^4^	4.448 × 10^2^	1.268 × 10^2^	1.151 × 10^3^	3.122 × 10^2^	4.851 × 10^2^

**Table 13:** The computation time(s) obtained by the algorithms for all images.

Images	*d*	ISMA	GWO	SOA	SMA	POA	MFO	× 10SMA	DFSMA
**Lena**	2	9.432 × 10^−1^	1.083	9.310 × 10^−1^	**9.043 × 10^−1^**	1.845	9.212 × 10^−1^	1.052	9.714 × 10^−1^
	3	9.787 × 10^−1^	1.010	9.458 × 10^−1^	9.425 × 10^−1^	1.884	**9.226 × 10^−1^**	1.065	9.793 × 10^−1^
	4	9.818 × 10^−1^	1.014	9.555 × 10^−1^	9.513 × 10^−1^	1.930	**9.239 × 10^−1^**	1.065	9.885 × 10^−1^
	5	9.949 × 10^−1^	1.035	9.718 × 10^−1^	9.809 × 10^−1^	1.935	**9.467 × 10^−1^**	1.083	9.954 × 10^−1^
**Cameraman**	2	9.312 × 10^−1^	9.974 × 10^−1^	9.064 × 10^−1^	9.085 × 10^−1^	1.809	**9.038 × 10^−1^**	1.021	9.622 × 10^−1^
	3	9.609 × 10^−1^	9.979 × 10^−1^	**9.065 × 10^−1^**	9.306 × 10^−1^	1.810	9.296 × 10^−1^	1.050	9.714 × 10^−1^
	4	9.830 × 10^−1^	1.019	**9.275 × 10^−1^**	9.616 × 10^−1^	1.821	9.818 × 10^−1^	1.099	1.005
	5	9.993 × 10^−1^	1.106	**9.597 × 10^−1^**	9.735 × 10^−1^	1.885	1.049	1.108 × 10	1.008
**Butterfly**	2	9.292 × 10^−1^	9.722 × 10^−1^	**8.903 × 10^−1^**	9.061 × 10^−1^	1.824	9.025 × 10^−1^	1.019	9.628 × 10^−1^
	3	9.725 × 10^−1^	1.010	9.337 × 10^−1^	9.457 × 10^−1^	1.894	**9.186 × 10^−1^**	1.061	9.794 × 10^−1^
	4	9.738 × 10^−1^	1.040	9.438 × 10^−1^	9.513 × 10^−1^	1.899	**9.296 × 10^−1^**	1.081	9.913 × 10^−1^
	5	9.874 × 10^−1^	1.054	**9.387 × 10^−1^**	9.785 × 10^−1^	1.954	9.739 × 10^−1^	1.110	1.022
**Lake**	2	9.374 × 10^−1^	9.805 × 10^−1^	9.026 × 10^−1^	9.165 × 10^−1^	1.761	**8.677 × 10^−1^**	1.021	9.617 × 10^−1^
	3	9.579 × 10^−1^	1.008	9.268 × 10^−1^	9.344 × 10^−1^	1.853	**9.102 × 10^−1^**	1.066	9.665 × 10^−1^
	4	9.754 × 10^−1^	1.037	**9.450 × 10^−1^**	9.683 × 10^−1^	1.935	9.566 × 10^−1^	1.076	1.004
	5	1.012	1.082	**9.520 × 10^−1^**	9.858 × 10^−1^	2.038969	1.116	1.095	1.012
**Barbara**	**2**	9.268 × 10^−1^	9.912 × 10^−1^	**9.002 × 10^−1^**	9.151 × 10^−1^	1.795	9.267 × 10^−1^	1.012	9.666 × 10^−1^
	**3**	9.520 × 10^−1^	9.994 × 10^−1^	**9.110 × 10^−1^**	9.322 × 10^−1^	1.825	9.289 × 10^−1^	1.043	9.723 × 10^−1^
	**4**	9.670 × 10^−1^	1.060	**9.269 × 10^−1^**	9.410 × 10^−1^	1.874	9.637 × 10^−1^	1.077	9.737 × 10^−1^
	**5**	9.669 × 10^−1^	1.067	1.008	**9.658 × 10^−1^**	1.958	1.014	1.078	9.918 × 10^−1^
**Columbia**	**2**	8.669 × 10^−1^	9.080 × 10^−1^	**8.273 × 10^−1^**	8.429 × 10^−1^	1.695	8.309 × 10^−1^	9.612 × 10^−1^	9.019 × 10^−1^
	**3**	8.855 × 10^−1^	9.397 × 10^−1^	8.683 × 10^−1^	8.592 × 10^−1^	1.724	**8.453 × 10^−1^**	9.789 × 10^−1^	9.080 × 10^−1^
	**4**	9.148 × 10^−1^	9.905 × 10^−1^	8.813 × 10^−1^	8.814 × 10^−1^	1.784	**8.741 × 10^−1^**	1.000	9.177 × 10^−1^
	**5**	9.216 × 10^−1^	1.004	**8.819 × 10^−1^**	9.072 × 10^−1^	1.791	8.966 × 10^−1^	1.034	9.359 × 10^−1^
**Milkdrop**	**2**	9.327 × 10^−1^	9.947 × 10^−1^	9.239 × 10^−1^	9.098 × 10^−1^	1.852	**9.218 × 10^−1^**	1.034	9.662 × 10^−1^
	**3**	9.550 × 10^−1^	1.028	9.331 × 10^−1^	**9.259 × 10^−1^**	1.882	9.569 × 10^−1^	1.031	9.668 × 10^−1^
	**4**	9.775 × 10^−1^	1.077	9.535 × 10^−1^	**9.528 × 10^−1^**	1.888	9.570 × 10^−1^	1.065	9.830 × 10^−1^
	**5**	1.005	1.064	**9.572 × 10^−1^**	9.829 × 10^−1^	1.964	9.628 × 10^−1^	1.081	1.007
**Man**	**2**	9.250 × 10^−1^	9.722 × 10^−1^	**8.956 × 10^−1^**	9.013 × 10^−1^	1.798	8.896 × 10^−1^	1.003	9.502 × 10^−1^
	**3**	9.755 × 10^−1^	1.028	**9.231 × 10^−1^**	9.396 × 10^−1^	1.876	9.378 × 10^−1^	1.052	9.905 × 10^−1^
	**4**	9.901 × 10^−1^	1.059	**9.410 × 10^−1^**	9.682 × 10^−1^	1.904	9.531 × 10^−1^	1.086	1.002
	**5**	9.974 × 10^−1^	1.163	**9.548 × 10^−1^**	9.836 × 10^−1^	1.942	9.572 × 10^−1^	1.097	1.012

## Data Availability

The data presented in this study are available on request from the corresponding author.
